# Antimicrobial Peptides as Cross-Seeding Modulators at the Neurodegenerative–Infectious Interface

**DOI:** 10.34133/research.1149

**Published:** 2026-02-24

**Authors:** Yanxian Zhang, Yijing Tang, Lijin Wang, Jie Zheng

**Affiliations:** ^1^ Division of Endocrinology and Diabetes, Department of Pediatrics, School of Medicine Stanford University, Stanford, CA 94305, USA.; ^2^ Department of Neurology Massachusetts General Hospital, Harvard Medical School, Boston, MA 02129, USA.; ^3^ Department of Biomedical Engineering and Chemical Engineering, The University of Texas at San Antonio, San Antonio, TX 78249, USA.

## Abstract

Antimicrobial peptides (AMPs), traditionally regarded as innate immune effectors, are increasingly recognized for their structural and functional convergence with pathogenic amyloids. Recent studies—including our own—reveal that AMPs not only exhibit antimicrobial activity but also modulate amyloid aggregation by accelerating, delaying, or redirecting fibril growth, acting at the nexus of protein misfolding, inflammation, and host defense. In this account, we highlight the emerging role of AMPs as cross-seeding modulators that can inhibit or promote amyloid fibrillization depending on structural context. We summarize mechanistic insights into how β-sheet-rich AMPs engage amyloidogenic targets via structural compatibility, directional seeding asymmetry, and surface-mediated catalysis. We also explore how AMP–amyloid cross-seeding contributes to a bidirectional pathogen–amyloid feedback loop, linking microbial infections to chronic inflammation and neurodegeneration. Building on these molecular foundations, we present recent design advances in engineering AMP-derived inhibitors with enhanced amyloid specificity, proteolytic stability, and translational potential. These dual-function peptides—capable of suppressing amyloid aggregation and modulating immune responses—offer a unique therapeutic strategy for diseases such as Alzheimer’s, type 2 diabetes, and systemic amyloidosis. We conclude by outlining current challenges and future directions for data-driven design, delivery optimization, and clinical development of multifunctional AMPs as next-generation therapeutics.

## Cross-Seeding as a Central Mechanism in Neurodegenerative and Infectious Pathologies

**Fig. 1.**
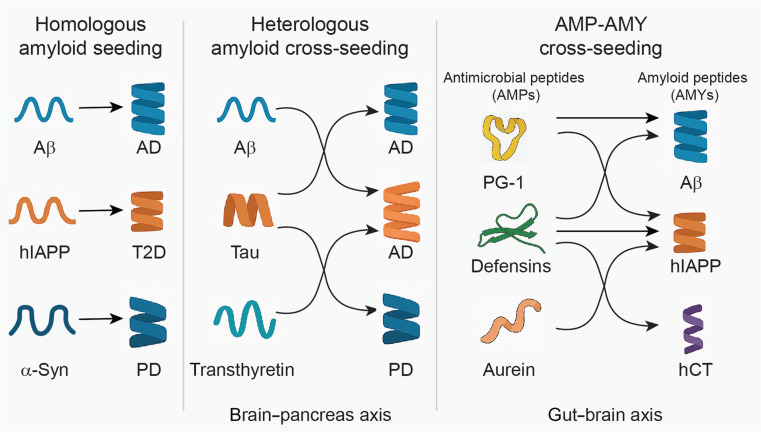
Conceptual overview of cross-seeding mechanisms in neurodegenerative and infectious pathologies, including homologous amyloid seeding, heterologous amyloid cross-seeding, and AMP–AMY cross-seeding.

Neurodegeneration and microbial infection, long considered distinct pathological processes, are increasingly recognized to share certain converging molecular mechanisms [1,2]. One key point of intersection is the phenomenon of cross-seeding—the ability of structurally compatible peptides to nucleate or modulate each other’s aggregation through specific interactions, most notably via β-sheet-based association [3]. Originally described among homologous amyloidogenic proteins, cross-seeding is now implicated in heterologous systems spanning host–pathogen interfaces, innate immunity, and neuroinflammation [1]. A conceptual overview of these cross-seeding paradigms—including homologous amyloid aggregation, heterologous amyloid aggregation, and antimicrobial peptide–amyloid peptide (AMP–AMY) crosstalk—is presented in Fig. 1, which highlights their potential roles in disease propagation across neurodegenerative, metabolic, and infectious axes. This mechanism provides a powerful conceptual framework to understand how immune response and protein misfolding may be mechanistically linked, especially in complex disease axes such as the gut–brain connection in Parkinson’s disease (PD) [4], or the increased comorbidity between Alzheimer’s disease (AD) and type 2 diabetes (T2D) [5]. These observations suggest that interclass molecular recognition, including that between antimicrobial and amyloid peptides, may represent a broader pathogenic principle across seemingly unrelated disorders.

### Homologous and heterologous amyloid seedings

Starting from amyloid seeding as the fundamental mechanism of protein aggregation, this process is pathologically associated with a broad class of protein misfolding diseases (PMDs). Many homologous amyloidogenic peptides, including amyloid-β (Aβ) in AD, human islet amyloid polypeptide (hIAPP) in T2D, and α-synuclein in PD, have the intrinsic ability to self-assemble into β-sheet-rich fibrillar structures through nucleation-dependent polymerization [6]. Extensive studies have elucidated the structural transitions, aggregation kinetics, and cytotoxic intermediates during this homologous amyloid seeding process [7–9]. The pathological cascade initiated by homologous amyloid seeding is central to the onset and progression of many PMDs. As these misfolded proteins accumulate, they disrupt cellular proteostasis [10], interfere with normal biochemical signaling [11], and initiate a range of deleterious downstream effects, including oxidative stress, mitochondrial dysfunction, and, ultimately, cell death [12,13].

Beyond homologous amyloid seeding, growing evidence indicates that heterologous AMYs, associated with distinct PMDs, can cross-seed—that is, initiate or accelerate one another’s aggregation through β-sheet-mediated molecular recognition. This amyloid cross-seeding has been observed in several pairings, including Aβ and α-synuclein [14], Aβ and tau [15], Aβ and transthyretin (TTR) [16], Aβ and hIAPP [17–19], and hIAPP and insulin [20]. Such interactions, confirmed in vitro and confirmed in patient-derived tissues, suggest that different amyloidogenic proteins can act cooperatively to exacerbate aggregation, spread pathology, and enhance cytotoxicity across otherwise distinct disease pathways. Mechanistically, heterologous amyloid cross-seeding contributes to pathology propagation, enabling misfolded amyloid species to transmit between cells and tissues. For instance, Aβ–hIAPP heteroaggregates propagate via the autophagy–lysosomal pathway, seeding secondary aggregates in distal cells [21]. In terms of toxicity, hybrid β-barrel oligomers formed by Aβ and hIAPP exhibit synergistic membrane disruption, inducing oxidative stress in both neuronal and pancreatic β-cell populations [22]. Clinically, cross-seeding has been proposed as a molecular driver of comorbidity: epidemiological data show that individuals with T2D have a 2- to 3-fold increased risk of developing AD, potentially linked to the transport and interaction of hIAPP and Aβ across the brain–pancreas axis [23]. Together, these findings support the existence of a shared structural misfolding code, wherein cross-β interactions enable molecular crosstalk between the same and different amyloid species, which provide a powerful rationale to explore cross-seeding as a unifying mechanism—and therapeutic target—across neurodegenerative and metabolic disorders.

### Converging structures and functions of AMPs and AMYs: Molecular basis for interclass cross-seeding

AMPs and AMYs have historically been categorized by divergent biological functions: host defense versus protein misfolding. However, some share striking structural and physicochemical similarities that challenge this traditional dichotomy. AMYs such as Aβ, hIAPP, α-synuclein, and serum amyloid A (SAA) are known to form β-sheet-rich fibrillar aggregates that drive cytotoxicity and organ dysfunction. AMPs, though generally shorter (10 to 40 residues), amphipathic, and cationic, have also been shown to adopt β-hairpin or β-sheet structures, particularly in membrane-like environments [24]. Protegrin-1 (PG-1), uperin 3.5, dermaseptin S9, and α-/β-defensins are notable examples of AMPs capable of forming stable or inducible β-structured aggregates, sometimes even assembling into amyloid-like fibrils [25].

This structural overlap is mirrored by functional convergence. Several AMPs exhibit amyloid-like self-assembly under physiological or stress conditions. Uperin 3.5, for instance, forms cytotoxic cross-β fibrils that mimic the morphology and membrane-disruptive properties of pathogenic amyloids [26]. Conversely, multiple AMYs—such as Aβ [27], hIAPP [28], and α-synuclein [29]—have demonstrated broad-spectrum antimicrobial activity, disrupting bacterial membranes via mechanisms reminiscent of host-defense peptides. Aβ can bind microbial carbohydrates via its heparin-binding domains and insert into lipid bilayers through hydrophobic residues, suggesting that it may act as an evolutionarily retained innate immune effector [30]. This bidirectional mimicry, in which AMPs exhibit amyloidogenic properties and AMYs acquire antimicrobial functions, defines a shared structural–functional interface that supports molecular recognition and aggregation between the 2 peptide classes.

Building upon these observations, recent studies—including our own—have demonstrated that interclass cross-seeding between AMPs and AMYs is not only plausible but also experimentally validated [31–35]. Depending on structural compatibility and environmental context, AMPs can either inhibit or promote amyloid aggregation through specific β-sheet interactions. Our recent work adds mechanistic and quantitative insights into this process. For example, PG-1 binds Aβ seeds with low micromolar affinity, efficiently blocking fibril formation, disassembling mature fibrils, and reducing Aβ-induced cytotoxicity, all while retaining its broad-spectrum antimicrobial activity [31]. A similar sequence-independent inhibition across multiple amyloid targets—including hIAPP and human calcitonin (hCT)—was observed with human and rabbit α-defensins (HNP-1 and NP-3A), where the inhibitory effects stemmed from β-structure-guided interactions rather than sequence complementarity [33]. Intestinal defensins, including human α-defensin 6 (HD-6) and human β-defensin 1 (HBD-1), further exemplify this dual functionality, demonstrating broad anti-amyloid activity across multiple peptide systems while maintaining bactericidal function under gut-relevant conditions [35]. Remarkably, these inhibitory effects were achieved at substoichiometric concentrations, indicating their high specificity and efficacy. However, cross-seeding is not inherently inhibitory. In contrast to the above, we found that aurein, a 13-residue AMP from *Litoria aurea*, promotes hIAPP fibrillization through β-sheet alignment with preexisting hIAPP seeds [32]. Yet, this enhancement of aggregation paradoxically coincided with a reduction in cellular toxicity, indicating that conformational compatibility can support either toxic or protective aggregation pathways, depending on sequence features and concentration ratios.

Other independent studies further support the mechanistic diversity and universality of cross-seeding. Bacterial amyloid proteins such as CsgA (from *Escherichia coli*) and FapC (from *Pseudomonas aeruginosa*) have been shown to accelerate host amyloid aggregation, including that of Aβ and α-synuclein, through heterotypic seeding [36,37]. In murine models, curli-expressing *E. coli* enhanced α-synuclein deposition and neuroinflammation, whereas CsgC, a curli-specific chaperone, inhibited this cross-seeding process [38]. Viral proteins from HSV-1 and HHV-6 may similarly act as seed templates or facilitate amyloid precursor protein (APP) cleavage, contributing to elevated Aβ production and misfolding [39]. These examples collectively reveal a bidirectional cross-seeding landscape that spans endogenous–exogenous and immune–amyloid protein systems, reinforcing cross-seeding as a central mechanism at the intersection of infection, inflammation, and neurodegeneration.

**Fig. 2.**
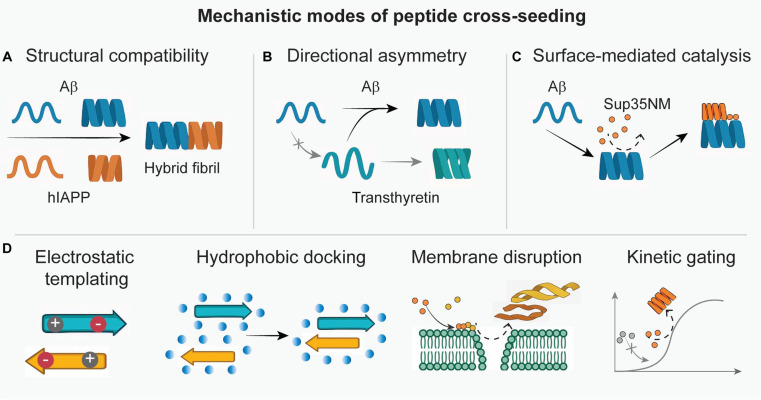
Mechanistic modes of peptide cross-seeding. Cross-seeding can proceed via (A) structural compatibility, where β-sheet-rich peptides co-assemble into hybrid fibrils; (B) directional asymmetry, where one peptide more efficiently seeds another; and (C) surface-mediated catalysis, where fibril or oligomer surfaces promote nontemplated aggregation. (D) Additional cross-seeding mechanisms include electrostatic templating, hydrophobic docking, and membrane-facilitated nucleation.

From a phenomenological standpoint, AMP–amyloid interactions fall into 3 primary mechanistic regimes. (a) In a disaggregation/remodeling mode, AMPs act as molecular detergents or end-cappers that bind preexisting oligomers or fibrils and convert them into alternative assemblies: they can fragment large fibrils, strip monomers from fibril ends, or reorganize oligomers into less ordered, more soluble complexes. (b) In a direct membrane-associated toxicity mode, AMPs and amyloid species independently or cooperatively disrupt cellular membranes—either by forming pores and carpet-like lesions or by thinning and destabilizing lipid bilayers—such that AMP binding to lipids, glycosaminoglycans, or receptors modulates how amyloids access and damage these surfaces. Here, AMPs can be cytotoxic, even in the absence of stable AMP–amyloid complexes, and amyloid aggregates may in turn sequester free peptide and thereby tune this activity. (c) In a cross-seeding/co-assembly mode, which is the focus of this review, AMPs and amyloid proteins are structurally integrated into joint, heteromeric assemblies rather than simply inhibiting or dissolving one another. This process generates hybrid oligomers or fibrils with toxicity profiles and stabilities distinct from the homotypic parent strains, acting as a “rheostat” that can either accelerate or suppress pathology depending on interfacial fit. In this regime, AMPs serve as structural templates, co-monomers, or surface catalysts that nucleate or accelerate aggregation of amyloidogenic partners, while concurrently reshaping oligomer–fibril equilibria and toxicity profiles. In real biological systems, these regimes frequently coexist and can be difficult to disentangle experimentally. An AMP that appears to “disaggregate” fibrils in bulk assays may in fact remodel them into off-pathway co-aggregates; an AMP that protects membranes at low concentration may, at higher concentration or in a different microenvironment, promote membrane-permeabilizing oligomers via cross-seeding. Distinguishing disaggregation/remodeling, membrane-centric toxicity, and cross-seeding/co-assembly as conceptually separate axes nevertheless provides a useful framework for interpreting apparently contradictory results and for organizing the case studies that follow. The “Microbial-Induced Amyloidosis: Pathogen–Amyloid Bidirectional Loop” and “Dual-Modulatory Role of AMPs” sections illustrate how specific AMPs traverse these regimes in disease-relevant contexts, whereas the “Central mechanisms of cross-seeding” section and Fig. 2 then dissect the molecular submechanisms of cross-seeding itself.

### Central mechanisms of cross-seeding

Cross-seeding between structurally ordered peptides—whether between homologous amyloids, heterologous amyloids, or distinct functional classes such as AMPs and AMYs—can proceed through multiple mechanistic pathways. These mechanisms differ in molecular detail but converge on a shared principle: structurally preorganized protein aggregates can nucleate or accelerate the misfolding and assembly of other peptides via physicochemical complementarity [3,40]. Three general classes of mechanisms are particularly relevant: structural compatibility, directional asymmetry, and surface-mediated catalysis.

Structural compatibility is often the most direct mechanism, wherein the cross-seeding efficiency is dictated by conformational alignment between seeds and incoming monomers (Fig. 2A). Even in the absence of strong sequence similarity, shared β-sheet motifs or supramolecular folding topologies enable stable co-assembly. For example, Aβ and hIAPP form hybrid fibrils through aligned U-bend β-strand geometries, stabilized by hydrophobic packing and interfacial salt bridges. Molecular dynamics (MD) simulations show that both peptides adopt convergent β-sheet topologies during heterotypic elongation, despite differing in primary sequence [19,41]. Similarly, defensins and PG-1 bind Aβ or hIAPP oligomers by inserting amphipathic β-structures into the growing aggregate core, effectively acting as “β-compatible wedges” that either disrupt or stabilize fibril growth [31,33].

Directional asymmetry is another defining feature of cross-seeding. Some peptide pairs show bidirectional seeding, where each species can catalyze the other’s aggregation (e.g., Aβ and α-synuclein) [42], while others exhibit unidirectional influence (Fig. 2B). For instance, TTR seeds promote Aβ aggregation, but the reverse is far less efficient, suggesting asymmetry in either structural compatibility or kinetic accessibility [42]. Similar asymmetries have been observed in AMP–AMY systems: aurein promotes hIAPP aggregation, but hIAPP has minimal effect on aurein’s assembly [32]. These examples underscore the importance of aggregation state specificity—monomer, oligomer, or fibril—as a determinant of seeding potential. Mechanistically, such directional asymmetry can arise from at least 3 nonexclusive sources: structural limitations, aggregation-state selectivity, and kinetic accessibility. Structurally, the fibril architecture or oligomeric surface of one partner may present grooves, charge patterns, or hydrophobic patches that are highly complementary to monomers or oligomers of the other, whereas the reciprocal assemblies do not provide an equivalently compatible interface. Aggregation-state selectivity adds another layer: seeds or oligomers of a given peptide may preferentially bind a specific conformer or oligomeric state of the partner that is rarely populated under the conditions used to probe the reverse direction. Finally, even when both directions are structurally allowed, the rate constants and nucleation barriers for cross-elongation, secondary nucleation, or fragmentation can differ by orders of magnitude, rendering one direction effectively silent on experimental timescales. Simple nucleation–elongation models extended to 2 interacting species reproduce this behavior: modest asymmetries in cross-elongation or secondary-nucleation efficiencies, or in fragmentation rates, are sufficient to yield strongly unidirectional seeding in global fits to kinetic traces. In AMP–amyloid systems, these considerations imply that apparent asymmetry (for example, aurein promoting hIAPP aggregation much more efficiently than the reverse) may reflect a combination of structural compatibility, preferred aggregation state, and differential kinetic accessibility, rather than a purely binary “on/off” cross-seeding relationship.

In many cross-seeding scenarios—particularly those involving unrelated or structurally mismatched peptides—surface-mediated catalysis emerges as a dominant pathway. Rather than integrating directly into the growing fibril via templated extension, surface-mediated catalysis, often described as heterogeneous or secondary nucleation, represents the nontemplated acceleration of aggregation via the physicochemical properties of the seed surface [43] (Fig. 2C). Typically, aggregation is initiated by small, aggregation-prone nuclei or soluble oligomers, which act as the earliest nanoscale seeds that concentrate monomeric substrates, align them locally, or perturb the surrounding hydration shell and electrostatic field—collectively lowering the energetic barrier for nucleation [6]. Importantly, such catalysis does not require sequence or structural homology between the seed and the recruited monomers, instead even structurally dissimilar peptides may catalyze each other’s aggregation through surface activity alone, especially when mediated by oligomeric interfaces with high local concentration and β-sheet propensity. A well-characterized example is the cross-seeding of Sup35NM, a yeast prion domain, by Aβ fibrils, despite the absence of sequence or structural homology [43]. In this case, the Aβ fibril surface functions as an amyloid-like nanoparticle, catalyzing aggregation via surface-mediated nucleation rather than templated elongation. The fibril’s physicochemical landscape, including surface charge, hydrophobicity, and curvature, facilitates the local organization of Sup35NM monomers into nucleation-competent assemblies. This surface-based mechanism is especially relevant to interclass cross-seeding systems such as AMP–AMY interactions. Here, peptides like PG-1 or α-defensins may not fully integrate into amyloid fibril cores, but still modulate aggregation behavior by presenting membrane-like or oligomer-mimetic surfaces that interfere with or accelerate nucleation [31,33,35]. As such, surface-mediated catalysis encompasses both mature fibrils and prefibrillar intermediates, expanding the definition of a “seed” and challenging classical models that rely solely on sequence or structural complementarity.

In addition to these dominant pathways, electrostatic templating, hydrophobic docking, and co-assembly through membrane disruption represent auxiliary mechanisms that modulate cross-seeding efficiency [19,44] (Fig. 2D). Charged peptides, for example, can engage in electrostatic prealignment, creating an energetically favorable interface for β-sheet zippering [6]. In AMP–AMY systems, these charge-mediated contacts often precede β-structural insertion [28]. Furthermore, some cross-seeding events are kinetically gated, where the aggregation of one peptide must reach a threshold oligomeric state to effectively seed another [45]. In a templated cross-elongation regime, the key parameter is the number of fibril ends: at fixed monomer concentration, increasing the number of seeds (for example, by fragmenting fibrils into shorter pieces at constant total mass) accelerates aggregation much more strongly than simply adding more unfragmented seed mass, and the reaction half-time scales approximately with the inverse of the end concentration. By contrast, secondary nucleation or surface-mediated catalysis is dominated by seed surface area and monomer concentration: aggregation rates show a steep, often higher-order dependence on monomer concentration, and the effect of added seeds can saturate once nucleation-competent surface is fully utilized. Practically, experiments that vary seed mass versus seed length and quantify how lag time or half-time changes with these variables provide a straightforward way to distinguish elongation-dominated from nucleation-dominated regimes. In AMP–amyloid systems, combining such seeding experiments with orthogonal readouts—fibril length distributions, end- versus surface-specific labeling, or 2-color cross-seeding assays—can help separate true cross-elongation (where AMPs are incorporated into the growing spine) from cases where AMP-decorated fibrils act primarily as catalytic surfaces for secondary nucleation.

Taken together, these mechanisms highlight the diverse and multivalent nature of cross-seeding, which encompasses not only sequence and structural complementarity, but also electrostatic prealignment, aggregation-state specificity, and interfacial catalysis. Cross-seeding thus emerges as a central mechanistic axis linking amyloidosis and microbial infection, with AMPs playing a uniquely versatile mediating role. By mimicking β-sheet structures or presenting membrane-like surfaces, AMPs can either antagonize or promote amyloid aggregation, depending not merely on sequence but also on secondary structure compatibility, membrane context, and dynamic binding equilibria. This dual functionality positions AMPs as compelling candidates for the rational design of cross-seeding modulators—peptides that not only disrupt pathogenic aggregation but also retain or enhance host defense activity. Understanding the molecular principles governing such interactions is critical for developing next-generation therapeutics and materials that operate at the intersection of protein misfolding, inflammation, and infection. In the following section, we examine how this molecular crosstalk plays out in physiological and pathological contexts, with particular focus on inflammation-driven amyloidosis and the bidirectional interactions between host peptides and microbial factors.

### Pathological and physiological implications of AMP–amyloid cross-seeding

Cross-seeding between AMPs and amyloidogenic proteins introduces a complex duality in which the same molecular interactions may either exacerbate disease pathology or contribute to host defense. The dual functionality of AMPs—as host-defense molecules and potential amyloidogenic agents—underscores a critical interface between immunity and protein misfolding [46]. Structurally, several AMPs share β-sheet (e.g., CsgA [36], CsgB, CsgC [38], CsgE [38], FapCS [37], and defensins [33,35]) or amphipathic α-helical motifs (e.g., magainin 2 [28], LL-37 [47], PSMα [48], and Lasioglossin-III [49]) similar to disease-associated amyloids, which enables them to interact, co-assemble, or nucleate amyloid formation through heterotypic seeding. From a pathological standpoint, this cross-seeding may exacerbate neurodegenerative conditions such as AD or PD by lowering the energy barrier for amyloid fibrillization [50]. Physiologically, however, certain AMP–amyloid interactions may serve beneficial roles, facilitating the sequestration of microbial toxins or modulating immune surveillance [30]. The context-dependent nature of these interactions—whether protective or pathological—may depend on concentration thresholds, peptide structure, posttranslational modifications, or the local inflammatory milieu.

#### Inflammation-induced AMP expression and pathological amyloid cross-seeding

In inflammatory settings, host tissues up-regulate AMPs as part of the innate immune response. However, in amyloid-prone environments such as the brain or pancreas, elevated AMP levels may inadvertently promote amyloidogenesis via cross-seeding. For example, the human cathelicidin LL-37 is markedly up-regulated during neuroinflammation and binds Aβ with high specificity [51]. While LL-37 can inhibit fibril formation by interfering with β-sheet adoption, thereby reducing long fibrillar aggregates characteristic of Alzheimer’s plaques, it paradoxically stabilizes soluble Aβ oligomers [47]. These oligomers are highly neurotoxic, particularly in the presence of activated microglia, where they stimulate pro-inflammatory cytokines such as tumor necrosis factor-α (TNF-α) and interleukin-6 (IL-6) [52]. This suggests that LL-37 may shift Aβ aggregation away from inert fibrils toward toxic oligomer retention, exacerbating neuronal injury. A similar mechanism is observed in T2D. In pancreatic islets, chronic inflammation increases levels of LL-37 and other AMP species, which bind hIAPP with nanomolar affinity [34]. While LL-37 suppresses hIAPP fibril elongation, it stabilizes toxic oligomeric intermediates that induce β-cell apoptosis through membrane disruption and mitochondrial dysfunction [53]. This mechanism may underlie the enhanced islet amyloid pathology and β-cell failure observed in diabetic patients with chronic infections or autoimmune comorbidities [54].

Emerging evidence also implicates other bacterial amyloids in this cross-seeding cascade. For example, α-synuclein, a presynaptic neuronal protein implicated in PD, can be cross-seeded by bacterial amyloids such as curli fibers produced by *E. coli* [36]. These interactions facilitate α-synuclein aggregation, contributing to neurodegeneration. Additionally, tau protein, associated with AD, can be cross-seeded by Aβ aggregates, promoting tau fibrillization and neurofibrillary tangle formation, further exacerbating neurodegenerative processes [55]. These interactions create a pathogenic feedback loop in which inflammation enhances AMP expression, which in turn promotes protein misfolding and aggregation, further sustaining local inflammatory damage.

#### Host defense and protein homeostasis: Dual roles of AMP–amyloid interactions

Beyond pathology, AMP–amyloid interactions may represent an evolutionarily conserved host-defense strategy. Several AMPs can form fibril-like assemblies that entrap bacterial or viral components, neutralizing pathogens through spatial sequestration or membrane disruption. These functional amyloids contribute to proteostasis and immune defense by serving as physical barriers or immune activators that enhance pathogen clearance, suggesting that cross-seeding is not inherently detrimental. Rather, it may reflect a fine-tuned balance between antimicrobial efficacy and protein homeostasis.

This duality is highly context-dependent. At physiological concentrations, LL-37 can inhibit fibril formation and neutralize the cytotoxicity of Aβ and hIAPP by stabilizing nonaggregating intermediates [34,47]. However, under chronic inflammation conditions, LL-37 expressions become dysregulated, leading to supraphysiological concentrations. In such environments, LL-37 can instead promote the formation of amorphous aggregates or stabilize membrane-disrupting oligomers [56], shifting the balance toward cytotoxic outcomes. Additionally, LL-37 not only modulates human amyloid proteins, but also inhibits the polymerization of bacterial amyloids, such as curli fibers formed by CsgA in *E. coli* biofilms [52]. Structural studies reveal that LL-37’s amphipathic helix binds to both bacterial CsgA [57] and human α-synuclein [58] via conserved hydrophobic motifs, enabling the formation of hybrid seeds. The hybrid seeds with bacterial curli proteins may propagate human amyloid aggregation and accelerate neurodegeneration, thereby linking microbial amyloids and host pathology through AMP-mediated crosstalk. Taken together, AMP–amyloid cross-seeding embodies a double-edged molecular mechanism—protective under regulated physiological conditions, but pathogenic when dysregulated by chronic inflammation or microbial insult. Maintaining this balance is critical to preventing chronic inflammation, proteostatic failure, and the onset of amyloid-associated diseases.

#### In vivo cross-seeding evidence and physiological constraints

Despite this growing mechanistic catalogue, truly in vivo evidence for AMP–amyloid cross-seeding remains sparse. Most of the reactions summarized in the “Cross-Seeding as a Central Mechanism in Neurodegenerative and Infectious Pathologies” and “Dual-Modulatory Role of AMPs” sections were defined in vitro using simplified buffers and micromolar peptide concentrations. By contrast, clinical cerebrospinal fluid (CSF) and brain-tissue studies indicate that classical AMPs such as LL-37, β-defensins, and liver-expressed AMP 2 are present in the central nervous system (CNS) at low-nanomolar to submicromolar levels under basal conditions [2,59,60], but can increase by 1 to 2 orders of magnitude in meningitis and severe neuroinflammation, and are detectable both in CSF and within neurons, glia, and barrier-forming cells of the meninges and blood–brain barrier (BBB). In AD, β-defensin-1 immunoreactivity is enhanced in hippocampal neurons and choroid plexus epithelium [59,61], and LL-37 expression is increased in human and mouse AD brains, where excess LL-37 drives microglial activation, promotes Aβ and tau deposition, and worsens cognitive decline [51,62]. Together with infection-driven models in which Aβ itself behaves as an AMP and deposits around bacteria or herpesviruses in mouse brain and 3-dimensional human neuronal cultures, these data demonstrate that innate immune peptides and amyloids do indeed encounter each other in vivo within physiologically plausible concentration ranges [27,30,39,46]. Moreover, BBB endothelial and meningeal cells up-regulate cathelicidin-family peptides (CRAMP/LL-37) in meningococcal meningitis, and neutrophil α-defensins can penetrate the BBB, indicating that both local synthesis and regulated barrier permeability can deliver AMPs into the parenchyma [63,64]. However, direct in vivo imaging or kinetic quantification of AMP–amyloid cross-seeding is still lacking, and we currently know little about how local microenvironments (membranes, extracellular matrices, and infection foci) concentrate AMPs above their bulk CSF levels.

In addition, AMP pharmacokinetics in the CNS impose important constraints on any attempt to translate cross-seeding concepts in vivo. Many cationic AMPs are short, protease-sensitive peptides that are rapidly degraded by extracellular peptidases, adsorbed onto cell surfaces and extracellular matrix, or cleared through CSF turnover and microglial uptake, making it challenging to sustain the prolonged micromolar exposures commonly used in vitro. Bridging this gap will require coupling CSF proteomics and spatially resolved peptide quantification with live-animal infection and aggregation models to test whether the in vitro cross-seeding reactions described here are quantitatively achievable in the intact brain, as well as systematic evaluation of AMP half-life and stability in brain interstitial fluid and CSF. From a design perspective, any therapeutic AMP-based cross-seeding modulator will need to balance chemical stabilization strategies (e.g., backbone cyclization, D-amino acid substitutions, and conjugation to carriers or nanoparticles) against preservation of the sequence and structural motifs that mediate the desired heterotypic interfaces.

## Microbial-Induced Amyloidosis: Pathogen–Amyloid Bidirectional Loop

Emerging evidence suggests a dynamic and reciprocal relationship between microbial infections and amyloidogenic pathways, implicating microbial agents not only as passive bystanders but as active modulators of amyloid formation [65]. This evolving paradigm introduces the concept of a pathogen–amyloid bidirectional loop, in which microbial components accelerate amyloid aggregation, while aggregated amyloids, in turn, amplify antimicrobial responses and neuroinflammation [1]. Here, we delineate the pathological and physiological implications of AMP amyloid cross-seeding, identify microbial triggers of amyloidogenesis, and conceptualize the feedback loop wherein inflammation and aggregation co-propagate, reinforcing a chronic disease trajectory.

### Microbial triggers of amyloid aggregation

**Fig. 3.**
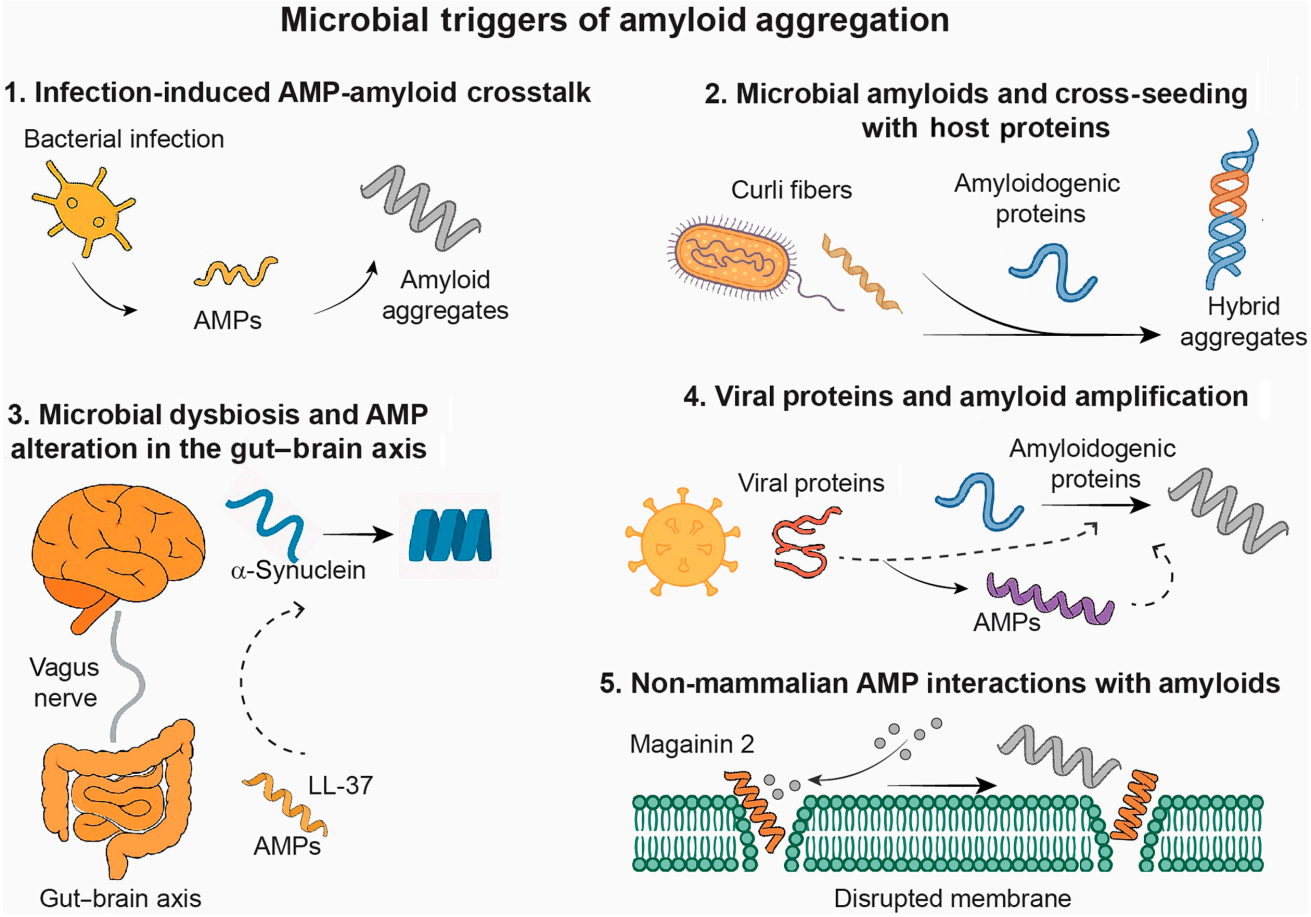
Schematic summary of microbial triggers and mechanisms underlying amyloid aggregation. The diagram illustrates 5 mechanistic categories: (1) Infection-induced AMP–amyloid crosstalk, where bacterial infections stimulate AMP release (e.g., CAP37, cathepsin G, and defensins), promoting or inhibiting amyloid aggregation; (2) Microbial amyloids and cross-seeding with host proteins, highlighting bacterial curli, viral proteins, and host AMPs contributing to hybrid aggregate formation; (3) Microbial dysbiosis and AMP alteration in the gut–brain axis, where gut-derived AMPs (e.g., LL-37 and defensins) and microbial amyloids influence α-synuclein misfolding and propagation; (4) Viral proteins and amyloid amplification, showing how viral proteins (e.g., SARS-CoV-2 spike and nucleocapsid) and eosinophil cationic protein (ECP) synergize in promoting amyloidogenesis; and (5) Nonmammalian AMP interactions with amyloids, including magainin 2-mediated membrane disruption and amyloid co-aggregation. Together, these pathways illustrate the multifactorial contribution of microbial stimuli and host peptides in modulating amyloid pathology.

Microbial agents, including bacteria and viruses, are increasingly recognized not only as potent inducers of inflammation but also as direct modulators of amyloidogenesis. Structural components such as lipopolysaccharides [66], outer membrane vesicles [67], and viral proteins [65] can serve as exogenous nucleation sites or destabilize host proteostasis, thereby promoting the aggregation of endogenous amyloidogenic proteins such as Aβ, α-synuclein, tau, and hIAPP. A key player in this process is the host’s AMP response, which is typically up-regulated during infection or inflammation. While AMPs are classically associated with innate defense, several also possess intrinsic amyloid-like properties or interact directly with misfolded proteins—exerting a range of effects from protective inhibition to pathological enhancement of aggregation. These distinct microbial influences and AMP-mediated pathways are summarized in Fig. 3, which outlines the mechanistic categories contributing to infection-associated amyloid propagation.

#### Infection-induced AMP–amyloid crosstalk

Bacterial infections are known to up-regulate AMP expression at inflammation sites, primarily through activation of innate immune signaling pathways. In addition to LL-37, several neutrophil-derived peptides—including CAP37, neutrophil elastase (NE), and cathepsin G—have demonstrated distinct anti-amyloid activities. CAP37 and NE inhibit the elongation phase of Aβ fibril growth, whereas cathepsin G primarily disrupts the nucleation step [68]. In parallel, human α- and β-defensins represent structurally conserved AMPs with β-sheet architecture stabilized by disulfide bonds. These peptides have been shown to bind to Aβ, hIAPP, and hCT, effectively preventing their fibrillization while preserving antimicrobial activity [33]. Mechanistically, these AMPs, released in abundance during microbial infiltration, appear to redirect amyloidogenic peptides away from fibril-forming pathways by stabilizing monomeric or early oligomeric intermediates. Such interactions likely reflect a conserved host defense mechanism for mitigating proteotoxic stress in response to infection. Their expression in inflammation-prone tissues—including cerebrovascular and peripheral compartments—positions these AMPs as critical modulators of infection-associated amyloidogenesis, particularly in the setting of infection-driven neuroinflammation and amyloid disease pathology.

#### Microbial amyloids and cross-seeding with host proteins

Beyond inducing AMP responses, many bacteria produce their own functional amyloids, with curli fibers from *E. coli* serving as a prototypical example. Composed primarily of the β-sheet-rich subunit CsgA, curli fibers have been shown to cross-seed the aggregation of human amyloidogenic proteins, including α-synuclein and Aβ, in both in vitro systems and animal models [36,69]. This cross-species interaction is influenced by the presence of host AMPs, which can either promote or inhibit hybrid aggregate formation depending on sequence compatibility and structural context. Accessory curli proteins such as CsgC and CsgE have also been implicated in modulating the nucleation efficiency and translocation of these aggregates across epithelial and neuronal interfaces [38,70]. Under inflammatory conditions, where AMPs like defensins and LL-37 are co-expressed, the formation of mixed microbial–host amyloid complexes may be further enhanced. These hybrid complexes often exhibit altered kinetics and increased cytotoxicity, serving as molecular bridges between localized infection and the systemic spread of amyloid pathology.

#### Microbial dysbiosis and AMP alteration in the gut–brain axis

The gastrointestinal tract represents a dynamic interface where host AMPs and the gut microbiota collectively regulate immune homeostasis and neural health. Under normal conditions, the expression of AMPs—such as defensins, RegIII family proteins, and cathelicidins—is tightly controlled to maintain barrier integrity and modulate microbial composition. However, during dysbiosis or chronic inflammation, this regulatory balance is disrupted, leading to altered AMP expression profiles and compromised mucosal defense [71]. Concurrently, certain bacterial populations, such as *E. coli* and *Salmonella*, may overproduce functional amyloids like curli fibers, which are capable of crossing epithelial barriers and interacting with host proteins [72]. Structural mimicry between microbial amyloids and neuronal proteins such as α-synuclein facilitates cross-seeding events that have been implicated in the initiation of α-synuclein aggregation within the enteric nervous system [50,58,70]. This process may be further modulated by host AMPs such as HBD-1 or LL-37, which can bind to both microbial amyloids and host-derived aggregation-prone peptides, stabilizing hybrid complexes with enhanced seeding capacity [35,52]. These AMP-mediated interactions may promote the prion-like transmission of misfolded α-synuclein from the gut to the brain, particularly along the vagus nerve, a route now increasingly recognized as a key conduit in amyloid disease pathogenesis [73]. Taken together, these findings underscore the importance of AMP–microbiota interactions not only in local immune defense but also in facilitating long-range amyloid propagation along the gut–brain axis.

#### Viral proteins and amyloid amplification

Viral infections, though typically acute, can exert lasting effects on host proteostasis by introducing aggregation-prone proteins and altering immune regulatory pathways, though the evidence varies across experimental scales. Biophysically, structural proteins from several viruses—including Herpes simplex virus [74], HIV [75], SARS-CoV-2 [76], and malaria [77]—contain sequence motifs that facilitate or accelerate the fibrillization of host amyloidogenic proteins in solution. In particular, in vitro kinetic assays have demonstrated that both the spike (S) and nucleocapsid (N) proteins of SARS-CoV-2 have been shown to promote the aggregation of Aβ and SAA, likely through a combination of electrostatic interactions, hydrophobic interfaces, and heparin-binding sequences embedded in their primary structures [78]. In the physiological environment, viral infections can dysregulate the expression of host-derived AMPs, notably eosinophil cationic protein (ECP), an arginine-rich peptide secreted in response to parasitic and viral pathogens. As a member of the RNase A superfamily, ECP accumulates in inflamed mucosal tissues, including the respiratory and gastrointestinal tracts, where it exhibits both antimicrobial activity and amyloid-like fibrillation under physiological conditions [79]. Its potential to interact with host amyloidogenic proteins and to disrupt cellular membranes suggests a mechanistic link between persistent infection, localized inflammation, and site-specific amyloid pathology. The co-occurrence of viral amyloid cofactors and AMP overexpression may synergistically enhance protein misfolding, particularly in mucosal environments where inflammation is prolonged. These molecular perturbations are especially relevant in aging or immuno-compromised individuals, in whom viral clearance may be delayed, leaving behind a pro-aggregatory milieu. However, translating these mechanistic observations to clinical pathology requires caution. While epidemiological studies strongly link chronic pathogens (e.g., HSV-1) to AD risk, evidence for acute viral triggers (like SARS-CoV-2) acting as direct amyloid accelerators remains heterogeneous. Current literature suggests a “bidirectional loop” where viral co-factors and AMP overexpression synergistically enhance misfolding, yet distinguishing direct viral cross-seeding from general inflammation-induced proteostasis failure remains a critical challenge for future in vivo validation.

#### Nonmammalian AMP interactions with amyloids

AMPs derived from nonmammalian species provide additional insight into amyloid-prone structures and infection-adaptive assembly. Several of these peptides exhibit pH- or stress-responsive self-assembly into amyloid-like structures, suggesting that infection-adaptive aggregation may serve functional roles beyond cytotoxicity. For example, dermaseptins such as PD-3-7, secreted by amphibians, form pH-sensitive fibrils under acidic conditions that mimic the microenvironment of infected tissues [80]. Similarly, insect-derived peptides including cecropin-C and lasioglossin LL-I show amyloid-like self-assembly behavior, although their capacity to cross-seed human amyloids remains to be fully elucidated [81]. In addition to structural self-association, some nonmammalian AMPs can interact synergistically with host amyloidogenic peptides. Magainin 2, a helical AMP from frog skin, has been shown to enhance membrane disruption when co-incubated with rodent islet amyloid polypeptide (rIAPP), suggesting a cooperative mechanism that amplifies membrane toxicity under metabolic or infectious stress [28]. This membrane-level synergy underscores the context-dependent nature of AMP–amyloid interactions, which may vary based on sequence compatibility, local concentration, and lipid composition. These peptides are typically released in large quantities during acute microbial challenges and exhibit membrane-disruptive activities similar to pathogenic amyloids. Their ability to transition into aggregated forms under environmental cues reinforces the concept that antimicrobial activity and amyloidogenic behavior can be co-encoded through evolutionary pressures. Although direct cross-seeding with human amyloids has not been extensively characterized, the structural plasticity and inducibility of these peptides make them compelling candidates for mediating cross-kingdom amyloid interactions, particularly at barrier sites such as the skin and gastrointestinal mucosa.

Together, these studies illustrate the multifaceted roles of microbial agents and AMPs in shaping amyloid aggregation pathways. While LL-37 remains a well-studied model, a wide array of AMPs—including defensins, proteolytic enzymes, eosinophil proteins, and nonmammalian peptides—exert diverse, context-dependent influences on protein misfolding. Their actions are shaped by sequence compatibility, local concentration, and tissue environment. Recognizing the breadth and specificity of these interactions is essential for understanding infection-induced amyloidogenesis and for identifying peptide-based therapeutic strategies to modulate pathogenic aggregation.

### Inflammation–aggregation feedback loop: A bidirectional axis

Across a wide spectrum of amyloid-associated diseases, accumulating evidence supports a bidirectional relationship between chronic inflammation and protein aggregation. Inflammatory signaling not only arises in response to misfolded protein accumulation but also actively promotes amyloidogenesis, forming a self-reinforcing cycle that drives disease progression [82]. Clinical observations support this model: in neurodegenerative conditions, neuroinflammation frequently coincides with—and may even precede—the development of pathological protein aggregates such as neurofibrillary tangles [83]. In peripheral inflammatory diseases like inflammatory bowel disease (IBD) and liver fibrosis, repeated cycles of immune activation and barrier disruption lead to tissue remodeling and stiffening, perpetuating inflammation in a manner that parallels the inflammation–aggregation axis [84,85]. These systemic and central parallels suggest that inflammation serves as both a sensor and an amplifier of proteotoxic stress, highlighting a shared pathogenic mechanism across multiple organ systems.

#### Amyloid aggregation triggers inflammation

With advancing age, neurons in the CNS progressively accumulate intraneuronal protein aggregates—many of which are not initially associated with disease. These aggregates, often structurally misfolded and insoluble, quietly build up over time and begin to trigger a low-grade but sustained inflammatory response, even in the absence of overt clinical pathology [86]. Accumulating evidence now supports that such misfolded protein aggregates are not merely passive by-products of aging or disease, but active instigators of innate immune activation [87]. Aggregates of Aβ, α-synuclein, and tau act as endogenous danger-associated molecular patterns (DAMPs), which are detected by pattern recognition receptors (PRRs) expressed on microglia, astrocytes, and peripheral immune cells [88].

Among these receptors, Toll-like receptors (TLRs)—especially TLR2 and TLR4—play central roles in sensing fibrillar Aβ and α-synuclein, leading to downstream activation of the NF-κB pathway and secretion of pro-inflammatory cytokines such as IL-1β and TNF-α [89]. In parallel, these aggregates also activate intracellular inflammasome complexes, most notably the NLRP3 inflammasome, which promotes caspase-1-mediated maturation of IL-1β and amplifies local inflammatory signaling [90]. Importantly, it is the β-sheet-rich fibrillar forms—rather than soluble monomers—that most potently elicit these innate responses, underscoring the immunogenic role of amyloid structure. The resulting neuroinflammatory environment not only recruits additional immune cells but also perturbs intercellular signaling, disrupts neuronal function, and accelerates neurodegeneration. Activated astrocytes release chemokines that attract monocytes and amplify cytokine cascades, while microglia may enhance Aβ production or promote tau hyperphosphorylation—thus feeding back into the amyloidogenic cycle [91]. These glial responses contribute to BBB breakdown, reactive oxygen species (ROS), and nitric oxide (NO) production, compounding the inflammatory burden [82].

**Fig. 4.**
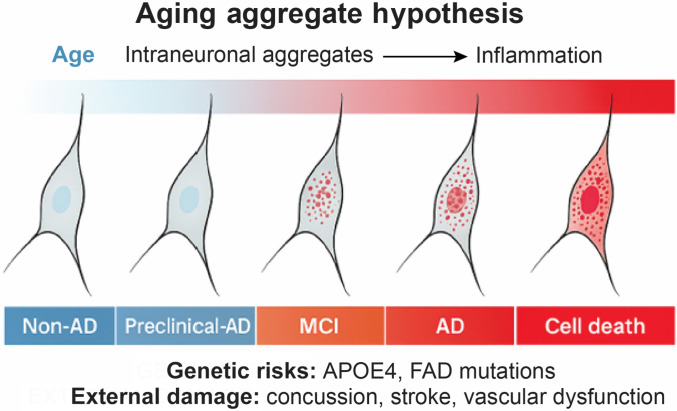
Aging-associated amyloid accumulation and inflammatory activation. Misfolded protein aggregates accumulate progressively in CNS neurons with age, initiating low-grade inflammation. This is exacerbated by genetic and environmental risk factors, leading to neuronal dysfunction and degeneration. The schematic illustrates the transition from preclinical accumulation to advanced Alzheimer’s pathology. This figure is adapted from a conceptual model originally published in Ref. [86]. MCI, mild cognitive impairment; FAD, familial Alzheimer’s disease.

This mechanism is not restricted to CNS. In peripheral tissues, similar cycles are observed. For example, in AA amyloidosis, aggregates of SAA accumulate in the liver, kidney, and spleen, where they provoke chronic local inflammation [92]. Additionally, AMPs such as LL-37 and ECP can themselves form amyloid-like fibrils that activate immune pathways via membrane disruption or TLR engagement [56,93]. Even microbial amyloids, such as curli fibers secreted by *E. coli* in gut biofilms, have been shown to activate host immune systems and trigger neuroinflammation, suggesting that the immune system recognizes β-sheet amyloid conformations—regardless of their origin—as a conserved signal of cellular danger [69]. Over time, the sustained burden of protein aggregates—compounded by genetic risk factors (e.g., APOE4 and familial AD mutations) and external insults such as stress, concussion, or vascular dysfunction—may drive neurons toward irreversible degeneration and cell death [86]. Collectively, these findings establish amyloid aggregates as potent immunomodulatory agents that activate PRRs, inflammasomes, and glial responses, creating a pro-inflammatory environment that not only contributes to disease progression but may also reinforce further aggregation. This conceptual pathological progression, from aggregate buildup to inflammation and ultimately to neuronal loss, is illustrated in Fig. 4.

#### Inflammation promotes protein aggregation

Chronic inflammation is increasingly recognized not only as a downstream consequence of protein misfolding but also as a potent upstream driver of amyloid aggregation. Once innate immune cells—such as microglia and astrocytes—are activated by amyloidogenic peptides like Aβ, they can themselves become sources of pro-aggregatory stimuli. Activated astrocytes, for instance, have been shown to increase endogenous Aβ production, while reactive microglia may either attempt clearance or, paradoxically, contribute to further deposition depending on the stage of activation and disease progression [94,95]. A key mechanism involves the release of inflammatory mediators including TNF-α, IL-1β, interferon-γ, and ROS/NO, all of which perturb protein folding environments and promote amyloidogenesis [96]. Notably, microglial-derived interferon-induced transmembrane protein 3 (IFITM3) has been shown to modulate γ-secretase activity, increasing Aβ production and favoring pathogenic aggregation [97]. In parallel, iron release from activated immune cells and oxidative stress conditions further destabilize neuronal proteostasis [98].

Inflammation also impairs the proteolytic clearance mechanisms responsible for degrading misfolded proteins. Chronic cytokine exposure suppresses autophagy and lysosomal function, leading to intracellular retention of tau, α-synuclein, and Aβ oligomers [82]. These aggregation-prone intermediates feed back into the cycle of glial activation and cytokine release, reinforcing a vicious loop of neurotoxicity. In AD models, chronic inflammatory signaling has been shown to enhance both Aβ plaque deposition and tau hyperphosphorylation [99]. Microglia may amplify tau pathology by secreting proline-directed kinases or facilitating its neuronal uptake and trans-synaptic spread. Likewise, systemic inflammation—such as that seen in IBD—can remodel the extracellular matrix and epithelial stiffness, creating environments conducive to local misfolding and aggregation [100]. Such mechanical and immune dysregulation may underlie emerging links between chronic gut inflammation and neurodegenerative risk.

These mechanisms are not confined to the CNS. In systemic amyloid diseases like AA amyloidosis, inflammation-induced overproduction of SAA leads to its aggregation and deposition in peripheral tissues. SAA accumulation not only reflects ongoing inflammation but also actively contributes to cytokine production, immune cell recruitment, and membrane disruption, further perpetuating the inflammatory milieu [101]. Recent evidence has even demonstrated that certain AMPs can be caught in this feedback loop. Even AMPs like LL-37 may shift from protective to pro-aggregatory roles under inflammatory stress [102].

**Fig. 5.**
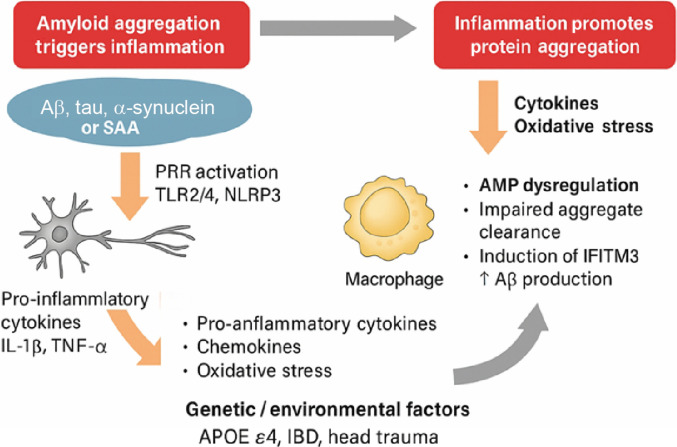
Inflammation–aggregation feedback loop in amyloid-associated pathologies. Misfolded protein aggregates activate innate immune pathways (e.g., TLRs and NLRP3), triggering cytokine release and glial activation. In turn, chronic inflammation promotes oxidative stress, alters protease activity, impairs amyloid clearance, and enhances amyloid production—forming a self-perpetuating cycle that drives neurodegeneration. Targeting this loop through anti-inflammatory interventions or inhibition of aggregation pathways offers a dual therapeutic strategy for slowing disease progression.

Together, Fig. 5 schematically illustrates the self-amplifying inflammation–aggregation loop that underlies both neurodegenerative and systemic amyloid diseases. The 2 reciprocal processes—amyloid-driven inflammation and inflammation-induced aggregation—form a self-reinforcing feedback loop central to both neurodegenerative and systemic amyloid pathologies. Inflammation is not merely a downstream response but also a primary accelerant of protein misfolding and aggregation. This cyclical axis highlights the therapeutic potential of concurrently targeting immune activation, oxidative stress, and proteostasis disruption to break the loop and mitigate disease progression.

### Clinical evidence: AMPs in human amyloid pathology

While in vitro and animal models establish the mechanistic plausibility of AMP–amyloid cross-seeding, the clinical relevance of these interactions must ultimately be grounded in human samples. Histopathological and biomarker studies are beginning to provide such evidence, although coverage is still uneven across diseases and cohorts.

In AD, the interplay between neuroinflammation and protein aggregation is well documented. Neuropathological analyses of postmortem brain tissue show that cathelicidin (CAMP) and β-defensin transcripts and proteins are enriched in regions of active pathology, including choroid plexus epithelium, hippocampal neurons, and plaque-adjacent glia, compared with age-matched controls [2,59,60]. Immunohistochemical and biochemical studies report robust β-defensin-1 immunoreactivity in these barrier and neuronal compartments, together with elevated LL-37 in human and mouse AD brain, consistent with local activation of innate immune defenses in neurodegenerating regions [51]. In parallel, fluid-biomarker work suggests that components of the AMP response are detectable systemically: several cohorts have described altered concentrations of β-defensins, neutrophil α-defensins (HNP1 to 3), and LL-37 in CSF, serum, or saliva of AD patients relative to cognitively normal individuals, with higher LL-37 or defensin levels sometimes correlating with worse cognitive performance or faster decline [61,103,104]. These findings do not yet demonstrate direct AMP–amyloid cross-seeding in vivo, but they support a scenario in which classical AMPs occupy the same anatomical compartments as Aβ and tau at disease-relevant concentrations.

For PD and related synucleinopathies, clinical evidence is more fragmented but points in a similar direction. Proteomic and immunoassay studies have identified α-defensins and other neutrophil-derived peptides as candidate CSF or plasma biomarkers in subsets of patients [105], and defensins as well as LL-37 are constitutively expressed in gut and brain tissues that are vulnerable to α-synuclein aggregation [62]. Peripheral neutrophil activation and chronic low-grade inflammation correlate with disease severity in several cohorts, raising the possibility that systemic or locally produced AMPs could influence α-synuclein aggregation kinetics at mucosal surfaces or within the CNS [106]. Direct co-localization of specific AMPs with Lewy bodies in human tissue remains an area of active investigation, but available data are consistent with an inflammatory milieu in which AMPs, α-synuclein, and microbial products coexist [58].

Beyond classical neurodegeneration, systemic amyloid diseases also provide examples of AMP-like molecules embedded in human aggregates. In T2D, although it is formally classified as a metabolic disease, we consider it here primarily as a systemic amyloid disorder in which the hormone islet amyloid polypeptide (IAPP/amylin) aggregates and crosstalks with brain amyloids. IAPP is deposited as amyloid within pancreatic islets, and ex vivo analyses of human pancreas show that the extent of IAPP amyloid burden correlates with β-cell loss and metabolic severity [107]. In this sense, T2D occupies a metabolic–neurodegenerative interface: IAPP behaves as an AMP-like hormone with antimicrobial and immunomodulatory functions, and IAPP–Aβ/tau cross-seeding provides a direct mechanistic bridge between peripheral metabolic dysfunction and central protein misfolding. IAPP and related peptides display antimicrobial and immunomodulatory activities, placing them at the interface between host defense and protein misfolding. In dialysis-related amyloidosis (DRA), β₂-microglobulin (β₂m) fragments dominate the fibril core, but proteomic profiling of patient-derived deposits reveals a complex “interactome” that includes complement factors, apolipoproteins, and inflammatory proteins; in some cases, AMP-like fragments or host-defense peptides are detected among the co-deposited components [108]. Although their functional contribution is not yet clear, these findings reinforce the notion that inflammatory mediators and amyloidogenic proteins share the same microenvironments in human disease.

Collectively, these clinical observations support the anatomical and quantitative plausibility of AMP–amyloid interactions in the human host: canonical AMPs and AMP-like hormones are present in CSF, blood, mucosal secretions, and affected tissues at concentrations that can, at least transiently, reach the low-nanomolar to submicromolar regime used in many in vitro studies. At the same time, the available datasets are still limited in size, disease breadth, and quantitative standardization. Large, longitudinal cohorts with harmonized AMP measurements across CSF, plasma, and tissue, coupled to detailed amyloid imaging and pathology, will be required to rigorously test causal links and to determine whether AMPs can serve as robust diagnostic or prognostic markers rather than generic correlates of inflammation.

## Dual-Modulatory Role of AMPs

AMPs, as key effectors of the innate immune system, exhibit a remarkable dual-modulatory function at the intersection of host defense and protein homeostasis. Traditionally recognized for their broad-spectrum antimicrobial activities—including membrane disruption and immune signaling modulation—AMPs have more recently emerged as regulators of amyloidogenic processes. Beyond their bactericidal and immunomodulatory roles, several AMPs directly interact with amyloidogenic proteins such as Aβ, tau, α-synuclein, and hIAPP, either inhibiting or, in some cases, promoting aggregation [33,35,49]. This duality highlights their structural versatility and evolutionary adaptation to manage both microbial threats and protein misfolding. This section categorizes AMPs into functional subtypes, illustrating how they modulate microbial clearance and amyloid aggregation through distinct molecular mechanisms.

### LL-37: A prototypical dual modulator

LL-37, the only human cathelicidin, exemplifies the dual-modulatory role of AMPs. Structurally characterized by its amphipathic α-helical conformation, LL-37 exerts potent antimicrobial effects primarily through membrane insertion, causing disruption and subsequent microbial cell lysis. Additionally, LL-37 plays an essential role in immune modulation, neutralizing bacterial endotoxins, recruiting immune cells, and regulating cytokine production [109]. Recent evidence further highlights LL-37’s capability as a direct modulator of amyloidogenic pathways [27,53]. It interacts with Aβ peptides via specific hydrophobic and electrostatic interactions, effectively inhibiting fibril formation and mitigating associated neurotoxicity both in vitro and in transgenic mouse models [27,47]. Structural analyses indicate that LL-37 preferentially binds amyloidogenic peptides at monomeric and oligomeric stages, thereby preventing nucleation and elongation phases critical to fibrillogenesis. Moreover, LL-37 effectively counteracts microbial-induced amyloid aggregation, disrupting the pathological cascade linking microbial infections to neurodegeneration [46]. These multifaceted roles position LL-37 as a promising therapeutic candidate with dual functionality, bridging innate immunity and amyloid modulation to potentially ameliorate both infectious and amyloid-associated disorders.

### Defensins: Multifunctional peptides at the interface of immunity and amyloid regulation

Defensins are evolutionarily conserved cationic peptides categorized into α- and β-defensins based on disulfide connectivity and tissue localization. Both subtypes play central roles in innate immunity and have been increasingly recognized for their ability to modulate amyloidogenic processes [36].

α-Defensins, such as human neutrophil peptides (HNPs), are cationic host-defense peptides predominantly expressed in neutrophils and stored in azurophilic granules. Their antimicrobial activity is primarily mediated through electrostatic interactions with bacterial membranes, resulting in pore formation and cell lysis [110]. However, very few studies have investigated their potential role in modulating amyloid aggregation. A recent study introduced the “anti-amyloid and antimicrobial hypothesis”, demonstrating that HNP-1 and rabbit NP-3A possess general, sequence-independent inhibitory activity against multiple amyloidogenic proteins, including Aβ, hIAPP, and hCT [33]. These defensins, enriched in β-sheet structures, interact with amyloid monomers and oligomers, preventing their transition into toxic fibrils even at substoichiometric concentrations. Importantly, these peptides retain their intrinsic antimicrobial activity when complexed with amyloid species, supporting their potential as multifunctional agents capable of simultaneously mitigating microbial infections and amyloid-associated toxicity.

β-Defensins are expressed in epithelial and mucosal tissues in response to microbial stimuli and exhibit broad antimicrobial activities [111]. HBD-1, in particular, has been implicated in AD pathophysiology. Elevated HBD-1 levels have been observed in the choroid plexus and hippocampal neurons of AD patients, where it localizes to granulovacuolar degeneration structures [59]. Iron accumulation—a hallmark of AD—has been shown to induce HBD-1 expression, suggesting a link between innate immune activation and neurodegeneration [59]. Building on these observations, HBD-1, traditionally recognized for its antimicrobial role in mucosal immunity, can be repurposed as a broad-spectrum amyloid inhibitor with dual functionality [37]. Through a cross-seeding strategy, HBD-1 was shown to interact with 3 clinically relevant amyloidogenic peptides—Aβ, hIAPP, and hCT—to inhibit their aggregation at substoichiometric concentrations. HBD-1 suppresses amyloid fibril formation by binding preferentially to amyloid monomers and oligomers, interfering with their transition into β-sheet-rich structures. Meanwhile, HBD-1 retained its intrinsic antimicrobial activity when complexed with amyloid peptides and effectively reduced amyloid-induced cytotoxicity in both neuronal (SH-SY5Y) and pancreatic (RIN-m5F) cell models [35]. These findings establish β-defensins—like their α-defensin counterparts—as promising candidates for dual-function intervention strategies for simultaneously disrupting amyloidogenesis and combating microbial infection.

**Fig. 6.**
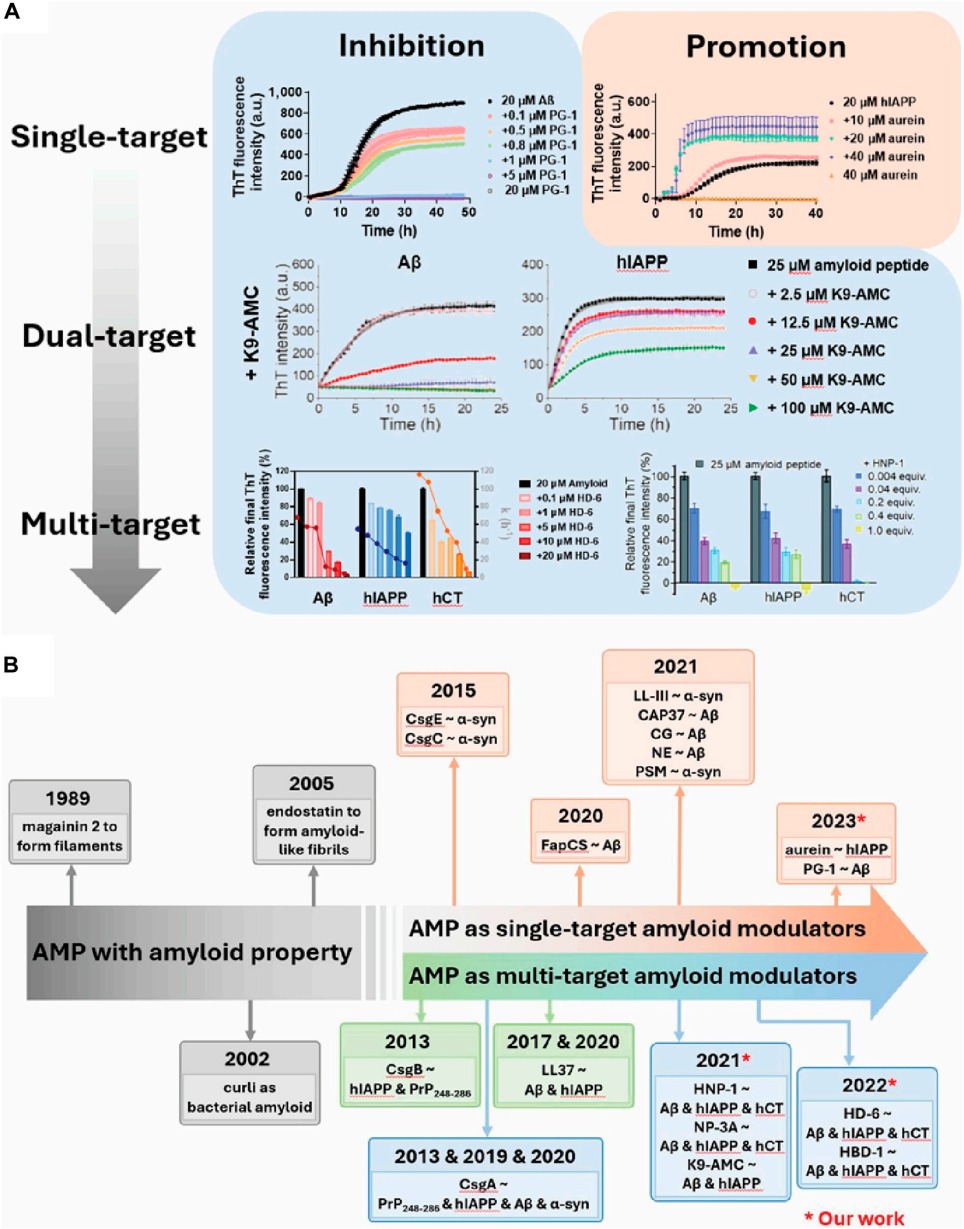
(A) Evolution of AMPs as amyloid modulators: from single-target to multi-target agents, which illustrates a conceptual framework for AMP-based inhibition of amyloid aggregation across increasing levels of target complexity. Top row (Single-target): Native AMP PG-1 demonstrates dose-dependent inhibition of Aβ fibrillization, while AMP aurein exhibits dose-dependent acceleration of hIAPP, as measured by Thioflavin T (ThT) fluorescence assays. Middle row (Dual-target): Rationally engineered AMP derivative K9-AMC concurrently inhibits aggregation of Aβ and hIAPP, indicating dual-target efficacy. Bottom row (Multi-target): Broad-spectrum peptides such as HD-6 and HNP-1 suppress fibril formation across 3 distinct amyloidogenic peptides (Aβ, hIAPP, and hCT). (B) Historical timeline of AMPs exhibiting amyloid-like properties or modulatory activity against amyloidogenic peptides. Early examples (1989 to 2005) include AMPs forming amyloid-like fibrils. From 2013 onward, increasing numbers of AMPs have been repurposed or discovered to inhibit aggregation of one or more amyloid targets (Aβ, hIAPP, and α-syn). The latest findings (highlighted in red) show rationally engineered or repurposed AMPs functioning as dual- or multi-target amyloid inhibitors via cross-seeding mechanisms (* indicates our work). In (A), the HNP-1-related bar chart is adapted from Ref. [33] under CC BY 3.0, while the HD-6-related multi-target inhibition data are adapted from Ref. [35] under CC BY-NC 3.0.

Despite their well-established antimicrobial roles, very few studies have explored α- and β-defensins as modulators of amyloid aggregation. Emerging evidence, including our recent findings, suggests that these conserved immune peptides possess untapped potential at the intersection of microbial defense and proteostasis. Their inherent multifunctionality—combining antimicrobial potency with sequence-independent inhibition of diverse amyloid species—underscores a promising yet underexplored therapeutic avenue. Future studies should prioritize mechanistic dissection and therapeutic development of defensin-based strategies targeting both infection and amyloidogenesis. This growing recognition of defensins’ dual functionality aligns with a broader conceptual shift in the field—namely, the evolution of AMPs from natural single-target inhibitors toward rationally designed agents with dual- and multi-target capabilities. This progression is illustrated in Fig. 6A, which highlights a conceptual framework for AMP-based amyloid modulation. At the single-target level, native peptides such as PG-1 demonstrate effective inhibition of Aβ aggregation in a dose-dependent manner [31]. In the dual-target tier, rationally engineered AMP derivatives like K9-AMC concurrently suppress aggregation of both Aβ and hIAPP, representing a substantial functional expansion [112]. At the multi-target level, broad-spectrum AMPs such as HD-6 and HNP-1 inhibit multiple amyloidogenic peptides—including Aβ, hIAPP, and hCT—demonstrating robust and versatile inhibition across distinct amyloid systems [33,35]. These findings underscore a functional trajectory from natural AMP activity to rationally optimized designs capable of targeting multiple amyloid species. This evolution reflects growing recognition of the shared structural motifs among amyloid proteins and the therapeutic need for agents that can modulate complex, multi-amyloid pathologies simultaneously. This progression is further illustrated in Fig. 6B, which presents a historical timeline of representative AMPs discovered as amyloid modulators—from early reports of AMP amyloid formation to the latest dual- and multi-target inhibitors identified through rational design and cross-seeding studies.

### Amyloidogenic AMPs: Functional overlap with pathological amyloids

While certain AMPs inhibit amyloid aggregation, a subset paradoxically displays intrinsic amyloid-forming capacity. These amyloidogenic AMPs self-assemble into fibrils that structurally resemble pathological amyloids, forming β-sheet- or α-sheet-rich architectures depending on environmental cues. This structural convergence between functional and pathological amyloids suggests an evolutionary overlap between microbial defense and protein misfolding.

**Fig. 7.**
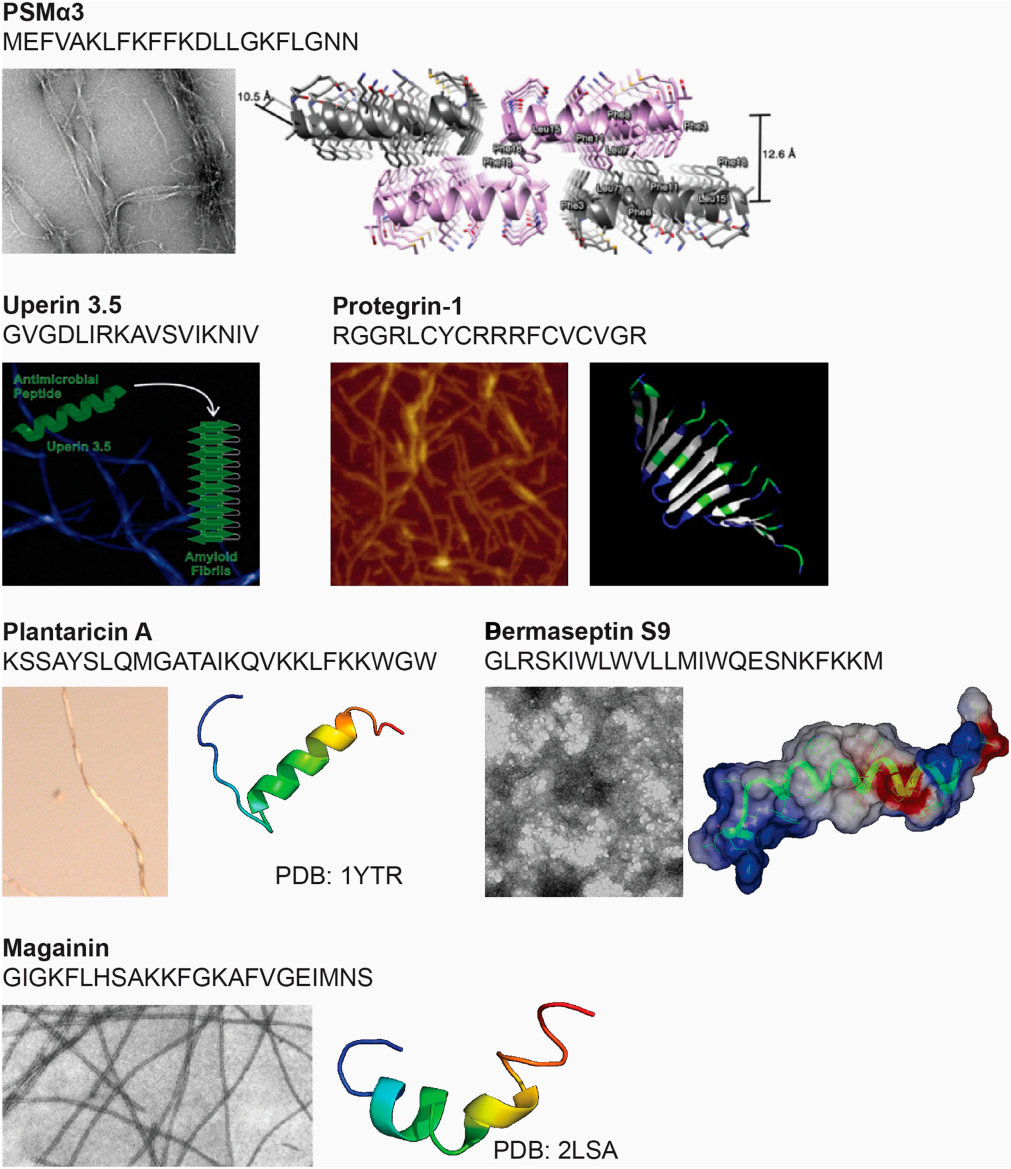
Representative amyloidogenic AMPs with demonstrated ability to self-assemble into fibril-like structures. Each panel presents the peptide name, amino acid sequence, and corresponding fibrillar morphology for PSMα3 [113], Uperin 3.5 [114], PG-1 [115], Plantaricin A [116], Dermaseptin S9 [118], and Magainin [117]. These AMPs highlight the structural diversity of innate immune peptides, including cross-α, cross-β, β-hairpin, and α-helical architectures, and exemplify their dual functionality in microbial defense and amyloid assembly. All images are reproduced from the cited references with permission from the copyright holders.

A well-characterized example is phenol-soluble modulins (PSMs) produced by *Staphylococcus aureus*. PSMα3 forms cross-α amyloid fibrils that reinforce bacterial biofilms and exert potent cytolytic activity against host membranes [113]. Despite their distinct fold, these assemblies can interact with host amyloids and may serve as cross-seeding templates. Similarly, the amphibian Uperin 3.5 demonstrates structural plasticity, adopting α-helical structures in antimicrobial states and transitioning to cross-β amyloids under aggregation-promoting conditions [114]. This conformational switch enables its dual role in defense and self-assembly. Several additional AMPs—including PG-1 [115], plantaricin A [116], magainin [117], and dermaseptin S9 [118]—have been shown to form amyloid-like fibrils with rapid kinetics. In Fig. 7, PSMα3 forms cross-α fibrils involved in biofilm stabilization; Uperin 3.5 adopts either cross-α or cross-β fibrils depending on context; Protegrin-1 forms β-hairpin structures stabilized by disulfide bonds; Dermaseptin S9 forms Congo red-positive β-sheet fibrils; and Plantaricin A adopts α-helical conformations with membrane-induced aggregation potential. These amyloidogenic AMPs retain or enhance their antimicrobial activity upon aggregation [26,81], indicating evolutionary co-option of aggregation-prone sequences for functional purposes.

There is increasing evidence that these peptides may also influence pathological amyloidogenesis via cross-seeding. Bacterial amyloids such as curli (CsgA) [36,48] and FapC [37] have been shown to accelerate the aggregation of α-synuclein and Aβ, offering mechanistic insights into how microbial infections may potentiate neurodegenerative disease. Despite these compelling observations, most studies remain descriptive, and systematic mechanistic investigations are still lacking. Future work should focus on elucidating the sequence–structure–function relationships of amyloidogenic AMPs and their interactions with host amyloid pathways. Insights from these naturally occurring AMPs not only clarify their dual roles in host defense and protein homeostasis but also inform the rational design of synthetic derivative for new therapeutic opportunities for disrupting amyloid propagation at the host–microbe interface.

### AMP-centric design principles of cross-seeding with amyloids

**Fig. 8.**
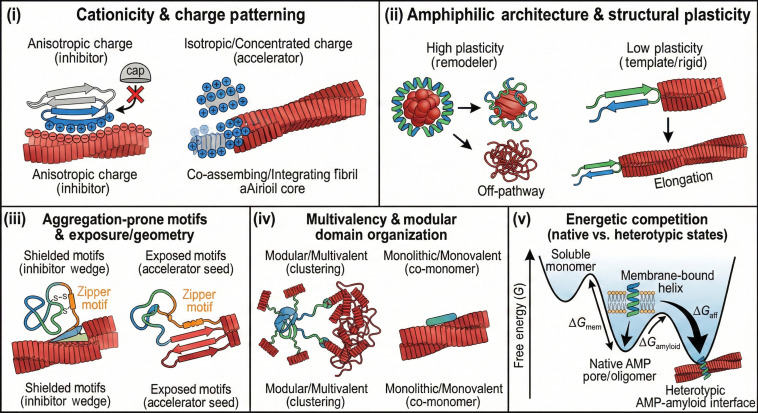
Antimicrobial peptide-centric design axes governing cross-seeding with amyloids, including (i) cationicity and charge patterning, (ii) amphiphilic architecture and structural plasticity, (iii) aggregation-prone motifs and their exposure/geometry in the AMP scaffold, (iv) multivalency and modular domain organization, and (v) energetic competition between heterotypic AMP–amyloid interfaces and native AMP.

The examples above show that AMPs do not interact with amyloidogenic proteins in an accidental, case-by-case fashion. Instead, their dual roles are encoded across multiple levels of organization—sequence, structure, conformational plasticity, multivalency, and energetics—as summarized schematically in Fig. 8.

#### Global cationicity and charge patterning

Most human AMPs fall within a relatively narrow window of positive net charge, but cross-seeding behavior is sensitive to the spatial distribution of cationic residues, not only to net charge. Enrichment of Arg/Lys in contiguous patches favors long-range electrostatic steering toward anionic amyloid surfaces (e.g., Aβ, hIAPP, and α-synuclein), while interspersed acidic residues or polar “spacers” can dampen this attraction and reduce nonspecific co-aggregation. For β-structured AMPs such as α- and β-defensins, clustering of cationic residues along one face of the β-sheet supports strong binding to negatively charged amyloid oligomers, yet the opposing face is dominated by more neutral or hydrophobic residues. This anisotropic charge distribution is consistent with their ability to bind multiple amyloid targets with minimal self-toxicity: one side of the peptide functions as a high-affinity docking interface, whereas the other side maintains solubility and prevents uncontrolled peptide–peptide stacking. By contrast, very short AMPs with highly concentrated positive charge and fewer polar residues are more prone to co-assemble with amyloid cores, which can tilt the balance toward aggregation-promoting cross-seeding.

#### Amphiphilic architecture and structural plasticity

AMPs are typically amphiphilic: hydrophobic residues segregate from polar/charged residues along helices, strands, or loops. This amphiphilic architecture, evolved for membrane binding and disruption, also allows AMPs to recognize the amphiphilic surfaces of amyloid oligomers and fibrils. A key discriminator between different cross-seeding regimes is structural plasticity. Helical AMPs (such as LL-37-like peptides) and β-rich AMPs (such as defensins) can redistribute their amphiphilic faces across multiple conformational states depending on environment—membrane, soluble oligomer, fibril, or condensate. This plasticity enables them to adapt binding modes: capping fibril ends, coating fibril surfaces, or bridging amyloid and membrane interfaces, often in a way that disfavors long-range, highly ordered peptide–peptide stacking. By contrast, some small amphibian or insect AMPs rapidly convert into rigid cross-β or cross-α assemblies upon binding amyloid surfaces; once locked into these architectures, their amphiphilic pattern becomes “hard-wired” into the fibril core, and they tend to promote templated elongation rather than dynamic capping or remodeling.

#### β-Aggregation hotspots and interface geometry

Embedded within the overall cationic–amphiphilic background, many AMPs (e.g., defensins, LL-37, aurein, and uperin) contain short stretches with elevated aggregation propensity—4 to 8 residue motifs enriched in hydrophobic and aromatic residues enriched in Val, Ile, Leu, Phe, and aromatic/polar pairs—that closely resemble canonical amyloid “zipper” motifs [47]. In β-structured AMPs, these motifs are often partially shielded within a disulfide-stabilized scaffold or loop, so that they can engage a partner without fully exposing a new amyloid-like spine. When such a motif docks against a preformed amyloid surface, it can act as a wedge or cap that perturbs the local packing of β-strands, slows elongation, or redirects monomers into off-pathway assemblies. In more flexible, helix–coil AMPs, aggregation-prone segments can transiently adopt β-like conformations at interfaces, stabilizing fuzzy hetero-oligomers or co-condensates that compete with ordered fibril growth. In contrast, when AMP hotspots are contiguous, solvent-exposed, and geometrically compatible with a given amyloid spine, the peptide can align in register with the partner’s backbone and extend the cross-β or cross-α lattice, accelerating fibril growth but potentially reducing the lifetime of small, highly toxic oligomers. Thus, not only the presence but also the placement and exposure of aggregation-prone motifs relative to the global scaffold shape whether an AMP behaves as an inhibitor, a conditional promoter, or a detoxifying “accelerator”.

#### Multivalency and modular domain organization

Many AMPs are functionally modular rather than monolithic. Folded cores, flexible tails, and oligomerization interfaces can combine to generate multivalent architectures that engage multiple amyloid partners simultaneously. Disulfide-stabilized β-sheet AMPs present repeated binding grooves; helical AMPs can oligomerize into bundles or rings; other cationic proteins with AMP-like domains present flexible basic tails attached to globular scaffolds. Such multivalency allows AMPs to cluster amyloid monomers, oligomers, or protofibrils into mesoscale assemblies that differ from the native fibril architecture. Depending on the balance of attractive and repulsive interactions, this can drive formation of off-pathway co-aggregates or condensates that reduce free monomer concentration, or it can create new high-avidity surfaces that catalyze nucleation. Short, monovalent AMPs lacking such modularity tend to behave more like single-site co-monomers in the fibril core, with less ability to reshape the mesoscale aggregation landscape.

#### Energetic competition with native AMP states

Superimposed on these structural axes is an energetic layer: AMPs must “choose” between several low-energy states—membrane-bound helices, soluble monomers, oligomeric pores, fuzzy co-aggregates, or incorporation into amyloid fibrils. Cross-seeding occurs when the free-energy gain from heterotypic AMP–amyloid interfaces is comparable to or greater than that of these alternative states. Strong membrane affinity, for example, can bias a cationic AMP toward remaining helical and surface-bound, even when the sequence contains β-prone motifs; in such cases, the peptide may primarily act as a scaffold that reorganizes local surfaces and ion distributions (a form of surface-mediated catalysis in the sense of the “Central mechanisms of cross-seeding” section), rather than entering the amyloid core. Conversely, when the energetic penalty for leaving the membrane is small and the heterotypic interface is highly favorable, the AMP is more likely to join chimeric fibrils or form stable co-aggregates.

Together, these AMP-centric axes (Fig. 8)—charge patterning, amphiphilic architecture and plasticity, motif exposure, multivalency, and energetic competition—define a design space in which natural and synthetic AMPs can be placed. β-rich, multivalent AMPs with partially shielded hotspots and anisotropic charge distributions tend to function as broad-spectrum inhibitors or remodelers of diverse amyloid families. Helical, conformationally adaptable AMPs occupy regions of the space that enable context-dependent modulation—capping some fibrils, clustering others into co-condensates, and bridging amyloid–membrane interfaces. Short, rigid, highly β-prone AMPs cluster toward a region where they behave as co-monomers and efficient cross-seeds, often increasing fibril mass while altering toxicity profiles. Formalizing this design space with quantitative descriptors (net charge and charge density, hydrophobic moment, β-propensity profiles, oligomerization tendencies, and membrane-binding energetics) will be essential for data-driven engineering of AMPs that deliberately avoid unwanted cross-seeding or, conversely, exploit heterotypic interactions to detoxify pathogenic assemblies or dismantle microbial amyloid scaffolds.

## Design Perspectives and Therapeutic Potentials of AMPs in Amyloid Modulation

While many AMPs adopt α-helical structures, a subset exhibits β-sheet-rich conformations, often stabilized by disulfide bonds, which confer structural rigidity and resistance to proteolytic degradation [24]. Interestingly, some β-sheet AMPs display amyloidogenic properties, forming fibrillar aggregates reminiscent of pathological amyloids observed in neurodegenerative diseases. This convergence suggests a nuanced interplay between antimicrobial defense mechanisms and amyloid pathophysiology. From a therapeutic design perspective, it is crucial to recognize that promoting fibrillization is not intrinsically beneficial or harmful, but strongly context-dependent. In systems where small, membrane-active oligomers are the dominant toxic species and fibrils are comparatively inert, accelerating fibril formation or redirecting aggregation into co-aggregates can be protective by depleting the oligomer pool. Conversely, in diseases where fibril burden, vascular deposition, or plaque-associated inflammation tracks more closely with pathology, further increasing fibril mass may be detrimental. Thus, AMP-derived modulators should be developed with an explicit, disease-specific choice between inhibiting aggregation and remodeling it into less toxic aggregates, rather than assuming that fibril promotion is universally desirable.

### Sequence–structure–function relationships of amyloidogenic AMPs: A missing link in data-driven discovery

While AMPs have long been studied for their pathogen-killing capabilities, a subset of AMPs exhibits amyloidogenic properties—forming β-sheet-rich fibrils that structurally resemble pathological amyloids. These dual-functional AMPs reside at a critical interface of host defense and protein misfolding disorders. However, the molecular determinants that underlie this dual behavior remain poorly characterized, particularly from a sequence–structure–function perspective. Machine learning (ML) and deep learning approaches have revolutionized AMP prediction and engineering. Models such as AMPlify [119], CAMP-R3 [120,121], and DeepAMP [122] use physicochemical features, sequence embeddings, and neural networks to predict antimicrobial potency, toxicity, or membrane interaction. Separately, the amyloidogenic potential of peptides has been modeled using tools like PASTA2.0 [123], AmyloGram [124], and Aggrescan [125], which rely on β-sheet propensity, hydrophobic clustering, and aggregation-prone motifs.

Despite advances in both AMP and amyloid prediction, no computational framework currently exists to identify or engineer AMPs with amyloidogenic or cross-seeding potential—a critical gap compounded by the limited understanding of their evolutionary origins, which could otherwise inform sequence–structure–function relationships and guide model development. The sequence–structure–function relationship of amyloidogenic AMPs can be elucidated through the integration of ML approaches with multi-dimensional peptide descriptors, including the following: (a) Curated datasets combining known amyloidogenic AMPs, such as LL-37, protegrins, and defensins, with conventional AMP datasets, such as Antimicrobial Peptide Database, Collection of Antimicrobial Peptides, Data Repository of Antimicrobial Peptides, and Database of Antimicrobial Activity and Structure of Peptides. (b) Transfer learning approaches leveraging pretrained models on AMP activity and fine-tuned on amyloidogenic labels. (c) Structure-aware models, including graph neural networks or AlphaFold-predicted conformations, to capture β-sheet motifs and aggregation hotspots. (d) Multi-objective optimization for designing AMPs with controlled antimicrobial and amyloidogenic balance, using reinforcement learning or generative models

In parallel, evolutionary features represent an underutilized yet informative design space. Two evolutionary routes are considered for the emergence of engineered AMPs. First, gene truncation events arising from mutations in longer coding sequences can yield bioactive peptide fragments. In a recent metagenomic analysis, over 50% of ortholog groups homologous to candidate AMPs were annotated as proteins of unknown function, consistent with previous microbiome studies [126]. Second, gene duplication followed by mutation may generate small, functional peptides, as observed in ribosomal proteins—some of which display both antimicrobial and amyloidogenic traits [127]. These dual characteristics suggest that amyloidogenicity may be an evolutionarily retained feature. Additional origins, such as horizontal gene transfer or expression from ancestral noncoding sequences, have also been proposed [128]. Incorporating these evolutionary patterns—via genome-context-aware embeddings, gene synteny, or orthologous group signatures—may enrich model inputs and support the discovery of novel dual-function AMPs.

Recent AI-driven AMP design platforms provide a natural starting point for extending “data-driven design” into the anti-amyloid space. Foundation-model-based frameworks such as AMP-Designer [129] already couple a large peptide language model to task-specific activity predictors and reinforcement learning to optimize antimicrobial potency, hemolysis, and plasma stability in an iterative design–test cycle, while DLFea4AMPGen [130] extracts key sequence fragments from deep-learning activity models and recombines them into multi-functional AMP candidates. Likewise, multi-objective generators such as MOFormer [131] and HMAMP [132] use conditional transformers or hypervolume-driven GANs, together with Pareto ranking of predicted minimum inhibitory concentration (MIC), hemolysis, and toxicity, to navigate trade-offs between efficacy and safety in silico. In principle, these architectures are agnostic to the choice of objectives: the same conditional heads or discriminator/predictor networks could be retrained on anti-amyloid endpoints (e.g., inhibition vs. promotion of aggregation, cross-seeding propensity, or toxicity of resulting aggregates), protease half-life, BBB penetration, and immune readouts (e.g., microglial activation and cytokine release), transforming them into platforms for multi-objective AMP–amyloid modulator design. However, current models inherit biases from their training data—most are trained on short, membrane-active, α-helical AMPs and property labels limited to MIC, hemolysis, and cell-level toxicity—so naïve use of their scores as proxies for anti-amyloid, CNS, or immunological behavior is not warranted. For the cross-seeding problem, retraining on curated datasets that explicitly couple AMP sequence and structure to amyloid-centric phenotypes, and prospectively validating model-generated peptides in standardized aggregation, proteolysis, pharmacokinetic, and immunological assays, will be essential to avoid overinterpreting in silico predictions and to realize the full potential of these tools for AMP-derived amyloid modulators.

### Rational design strategies for AMP-derived inhibitors for amyloid modulation

**Fig. 9.**
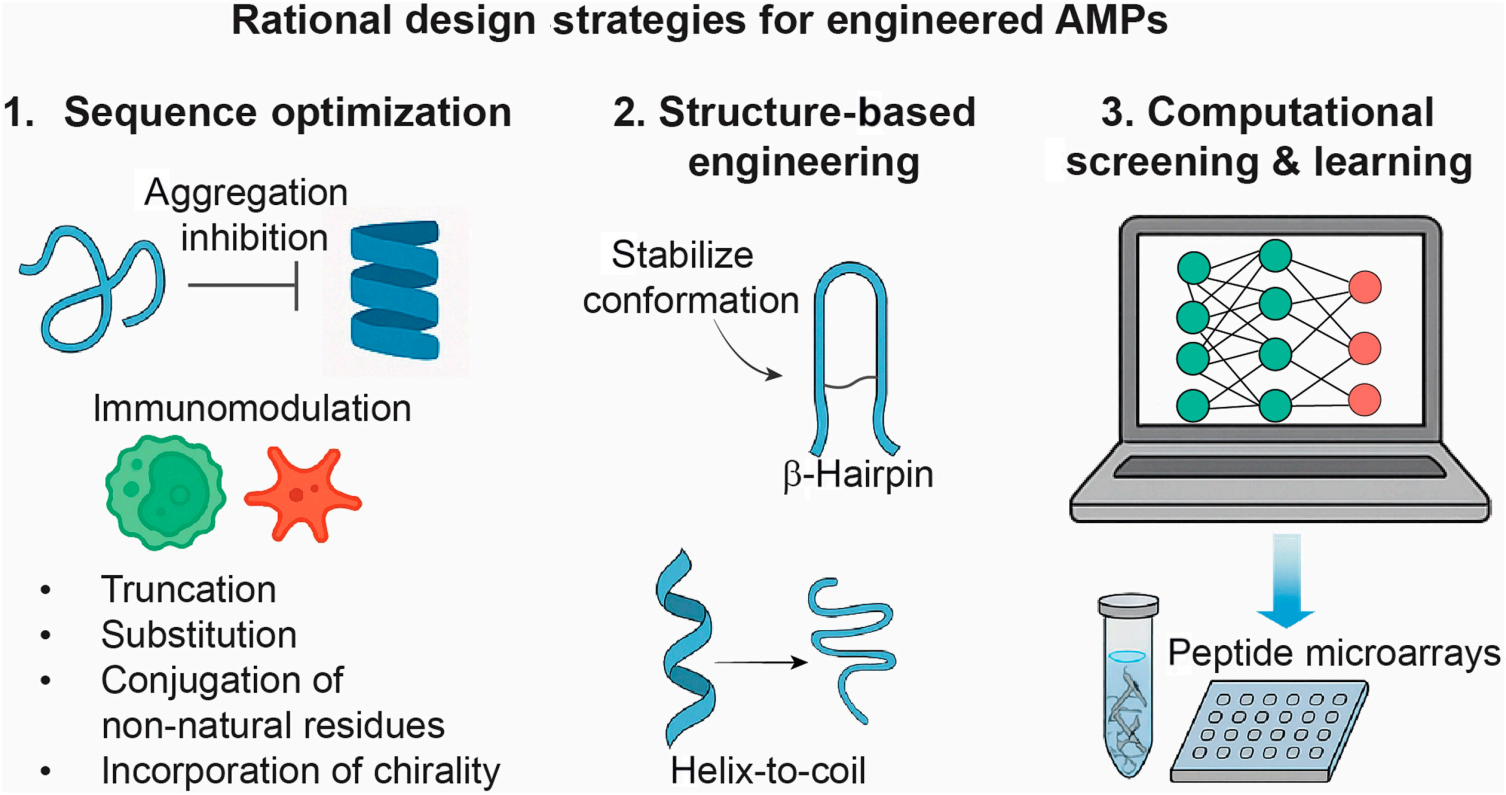
Overview of rational design strategies for engineered AMPs targeting amyloid fibrils. The design framework includes the following: (1) Sequence optimization, involving truncation, substitution, chirality incorporation, and nonnatural residue conjugation to enhance aggregation inhibition and immunomodulation; (2) Structure-based engineering, focusing on stabilizing peptide conformations (e.g., β-hairpins) and helix-to-coil transitions to achieve conformational specificity; and (3) Computational screening & learning, which utilizes machine learning models and peptide microarrays for in silico prediction and in vitro aggregation assays to evaluate inhibitory efficacy.

Building on the emerging understanding of AMPs as dual-function agents with both antimicrobial and amyloid-modulating activities, recent efforts have shifted toward the rational design of AMP-derived inhibitors that can specifically target amyloid aggregation pathways (Table 1). Unlike traditional drug discovery, which often screens large libraries of small molecules, AMP-based design leverages their inherent structural adaptability, membrane-binding capabilities, and innate immune signaling properties. These peptides can be systematically modified to optimize their anti-amyloid efficacy while minimizing cytotoxicity or immune activation. Current strategies for engineering AMP variants to enhance amyloid inhibition include sequence optimization, structural tuning, conjugation approaches, and computational screening (Fig. 9).

**Table 1.** Comparative inhibitory performance of various amyloid inhibitors

	No.	Inhibitor	Amyloid	Molar ratio ^a^	Cytotoxicity reduction	References
Sequence-specific inhibitors	1	Polyphenol phtalocyanine tetrasulfonate (PcTS)	α-syn	15	25%	*Nat. Comm.* 2014 [172]
	2	Cyclic D,L-α-peptides	Aβ	10	55%	*J. Am. Chem. Soc.* 2013 [173]
	3	Squalamine	α-syn	10	79%	*PNAS* 2017 [174]
	4	Carbazole-based fluorophore SLOH	Aβ	5	30%	*Angew. Chem.* 2012 [175]
	5	Tolcapone	TTR	5	50%	*Nat. Comm.* 2016 [176]
		Tafamidis		10	40%	
	6	Aminopyrazole trimer derivatives	Aβ	6	~100%	*J. Am. Chem. Soc.* 2011 [177]
	7	β casein-coated AuNPs	Aβ	2.5	69%	*Nat. Comm.* 2019 [178]
	8	Foldamers	Aβ	1	~100%	*J. Am. Chem. Soc.* 2017 [179]
	9	Pt(II)-1,10-phenanthroline complexes	Aβ	1	60–100%	*PNAS* 2008 [180]
	10	D-peptide	Tau	1	N/A	*Nature* 2011 [181]
	11	BRICHOS domain of Bri2	hIAPP	1	N/A	*PNAS* 2018 [182]
	12	Adapalene	Aβ	1	31%	*PNAS* 2017 [183]
	13	β-wrapin AS69	α-syn	1	83%	*Angew. Chem.* 2014 [184]
	14	Mimics of the IAPP cross-amyloid interaction surface with Aβ	Aβ	1	88%	*Angew. Chem.* 2015 [185]
	15	Amyloid β-sheet mimics ABSM 1a	Aβ	1	33%	*Nat. Chem.* 2012 [186]
		ABSM 1m	β_2_m	1	N/A	
		ABSM 1o	α-syn	0.5	N/A	
	16	Double N-methylated IAPP analog	hIAPP	1	77%	*PNAS* 2006 [187]
	17	VQIINK inhibitors	Tau	0.4	N/A	*Nat. Chem.* 2018 [188]
	18	Globular protein fused α-syn	α-syn	0.1	N/A	*Angew. Chem.* 2018 [189]
	19	Gammabody inhibitor	Aβ	0.1	~100%	*PNAS* 2012 [190]
	20	iAβ-H	Aβ	~1	45%	*PNAS* 2022 [191]
		iTau-P	Tau	>2	N/A	
		iα-syn-F	α-syn	1	44%	
	21	K3-L3-K3-GI	Aβ	1	N/A	*Angew. Chem.* 2021 [192]
	22	PG-1	Aβ	0.05	12%	*ACS Chem. Neurosci.* 2023 [31]
Dual inhibitors	23	Rhodanine-based molecule 1	hIAPP	>10	N/A	*Angew. Chem.* 2007 [193]
			Tau	>10	30%	
	24	Rhodanine-based molecule 2	hIAPP	>10	N/A	*Angew. Chem.* 2008 [194]
			Tau	1	31%	
	25	Macrocyclic peptides 1a	Aβ	1	80%	*Angew. Chem.* 2018 [195]
		2a		2	60%	
		1a	hIAPP	1	88%	
		2a		2	56%	
	26	Cucurbit[7]uril	Aβ	>10	50%	*Angew. Chem.* 2014 [196]
			Insulin	0.5	~100%	
	27	Cathelicidin	Aβ	1	86%	*J Alzheimers Dis.* 2017 [47]
	28	LL-37	hIAPP	1	55%	*Angew. Chem.* 2020 [34]
	29	N-methylated IAPP mimics	Aβ	1	42%	*Angew. Chem.* 2013 [197]
			hIAPP	1	55%	
	30	ACMs	Aβ	1	30–40%	*Nat. Chem.* 2022 [198]
			hIAPP	2–2.5	24–35%	
	31	K9-AMC	Aβ	2	22%	*Adv. Funct. Mater.* 2021 [112]
			hIAPP	>4	9%	
Broad-spectrum inhibitors	32	Lysine-specific molecular tweezers	TTR	10	~100%	*J. Am. Chem. Soc.* 2011 [199]
			β_2_m	10		
			hCT	10		
			hIAPP	1		
			Aβ	10		
			Tau	1		
	33	HNP-1, NP-3A	Aβ	1	23%	*Chem. Sci.* 2021 [33]
			hIAPP	1	28%	
			hCT	0.2	24%	
	34	HD-6, HBD-1	Aβ	1	52–55%	*Chem. Sci.* 2022 [35]
			hIAPP	>1	31–32%	
			hCT	1	7–18%	

α-syn, α-synuclein; Aβ, amyloid β-protein; TTR, transthyretin; hIAPP, human islet amyloid polypeptide; β_2_m, β_2_-microglobulin; hCT, human calcitonin

^a^ Molar ratio: molar ratio of inhibitor to amyloid peptide that completely suppress amyloid aggregation.

#### Sequence optimization

A fundamental strategy in the rational design of AMP-derived inhibitors is sequence optimization—modifying amino acid composition to enhance target specificity, aggregation modulation, and biocompatibility. Given the structural plasticity of many AMPs, even single-residue substitutions can markedly alter their folding behavior, aggregation propensity, or interaction with amyloid fibrils. One common approach involves truncation or domain-focused redesign to retain amyloid-binding motifs while minimizing off-target effects. For example, truncated fragments of LL-37, such as KR-12, have been shown to preserve anti-inflammatory and anti-aggregation activity while reducing cytotoxicity [133,134]. These shorter peptides maintain amphipathicity and cationic charge, facilitating selective binding to misfolded protein surfaces and microbial membranes. Additionally, amino acid substitution guided by aggregation predictors or β-sheet disruptors has been employed to design protegrin and defensin analogs with reduced amyloidogenicity. For instance, Protegrin-1 analogs with modified β-hairpin turns and cysteine spacing have demonstrated altered amyloid fibril formation while retaining antibacterial activity [31]. Chirality modifications, including the incorporation of D-amino acids, can also improve proteolytic stability and inhibit aggregation through steric interference. D-peptide versions of amyloid-binding sequences have been shown to block fibrillization in models of Alzheimer’s and prion diseases, although this strategy remains less explored for AMP-derived scaffolds [135]. Incorporating nonnatural residues or aggregation-disrupting motifs, such as proline or β-breakers (e.g., Aib [136] and GxxxG [137]), can further tune peptide conformation to prevent pathological self-assembly. For example, Pro-rich AMP analogs have been shown to interfere with Aβ aggregation and oligomerization without forming toxic intermediates [138]. Overall, sequence-level modifications provide a flexible design handle for tuning amyloid-interacting properties of AMPs. When guided by biophysical characterization and in silico predictions, these strategies enable systematic exploration of the sequence space toward optimized dual-function therapeutics.

#### Structure-based engineering

Beyond sequence-level modifications, structure-based engineering offers a robust strategy for optimizing AMP-derived inhibitors to target amyloid fibrils with molecular specificity. Many amyloidogenic peptides, including Aβ, α-synuclein, and hIAPP, form structurally ordered β-sheet-rich fibrils. These well-defined architectures provide steric and electrostatic interfaces that can be selectively targeted by peptides engineered with complementary structural motifs. One widely adopted approach is the design of β-hairpin or amphipathic α-helical conformations to selectively interact with amyloidogenic intermediates. Engineered β-hairpin peptides, often stabilized by disulfide bridges or macrocyclization, have been shown to effectively inhibit Aβ fibrillization by capping fibril ends or disrupting oligomer nucleation processes [139]. Such conformational restriction enhances structural recognition and binding affinity. Notably, rationally designed acyclic β-hairpin mimics, built on β-turn-inducing scaffolds, have been demonstrated to inhibit both oligomerization and fibrillization of amyloidogenic proteins such as hIAPP and Aβ [140]. These mimics act early in the aggregation pathway, interfering with primary nucleation events and delaying membrane leakage induced by toxic oligomers—an important mechanism in amyloid cytotoxicity. Additionally, HI18, an engineered β-hairpin-binding protein, was designed to recognize hIAPP in its β-hairpin conformation. Remarkably, HI18 inhibited IAPP aggregation and cytotoxicity at substoichiometric concentrations [141], suggesting the efficiency of conformation-specific inhibition. These studies collectively highlight the critical role of β-hairpin molecular recognition motifs in modulating amyloid aggregation pathways.

For β-sheet-rich AMPs, such as defensins, protegrins, and other β-hairpin or β-sheet miniproteins, structure-based engineering can leverage a more explicit set of physicochemical parameters that bias cross-seeding versus inhibition. Unlike flexible peptides that must pay a high entropic cost to adopt a specific conformation, these disulfide-stabilized scaffolds offer a preorganized template for interaction. Key tunable features include β-strand length and registry (which determine how easily an AMP segment can align in-register with a given amyloid spine), surface charge density and anisotropy (the distribution of Lys/Arg vs. acidic and polar residues across the β-sheet faces), and the placement of patterned hydrophobic and aromatic residues to create dry steric-zipper-like patches compatible with, or deliberately mismatched to, target amyloid segments. Disulfide topology and loop length further control scaffold curvature and hotspot exposure: more rigid, tightly cross-linked β-sheets can present aggregation-prone motifs as shallow surface grooves that cap or distort amyloid fibrils, whereas more flexible, partially exposed β-hairpins can insert as co-monomers into the growing cross-β lattice. Finally, self-association interfaces and oligomerization propensity set the degree of multivalency, which influences whether a β-sheet AMP primarily coats fibril surfaces, bridges multiple protofibrils into co-aggregates, or remains monomeric and acts as a stoichiometric end cap. In combination, these parameters provide a concrete design space for β-sheet-rich cross-seeding modulators: by tuning β-strand registry, charge patterning, hydrophobic patch geometry, and disulfide connectivity, one can bias a given scaffold toward fibril capping and remodeling versus heteromeric spine extension.

In parallel with AMP-derived scaffolds, several recent protein-assembly strategies provide complementary design concepts for amyloid modulation. Kong et al. [142] have shown that short, hydrogen-bond-driven N-terminal Aβ mimetics, designed to engage the His13–His14 boundary between disordered and ordered regions, can redirect Aβ42 aggregation, attenuate oligomer toxicity, and be formulated as ROS-responsive hydrogels for localized delivery. This “like-interacts-with-like” strategy—using β-prone peptide motifs that selectively recognize conformationally labile segments of Aβ rather than the fibril core—strongly resonates with AMP-inspired designs that target specific sequence and structural hotspots rather than acting as nonspecific detergents. Interfacial assembly studies further highlight how supramolecular context can be engineered to tune amyloid mechanics and adhesion. Thiol-regulated interfacial protein aggregation achieves living/controlled supramolecular polymerization of partially unfolded proteins at air–water or solid–water interfaces, producing nanofilms whose thickness grows linearly with monomer conversion and stepwise monomer feeding [143]. Related work with lysozyme-based amyloids shows that fibrils formed from full-length proteins, which retain α-helical and disordered segments around a β-sheet core, display markedly higher adhesion and viscoelasticity than peptide-fragment fibrils composed mainly of rigid β-sheets [144]. Nonfibrillar Janus amyloid nanofilms further demonstrate that asymmetric residue distribution at interfaces can yield exceptionally stiff and adhesive coatings [145]. Together, these systems illustrate how motif targeting, interfacial confinement, and controlled supramolecular polymerization can be combined with AMP-based sequence and structure principles to expand the design space for cross-seeding modulators and amyloid-inspired materials.

Helix-to-coil transitions represent another structural mechanism by which AMP-derived inhibitors can destabilize amyloidogenic interfaces. Peptides with inherent α-helical structures—such as LL-37 and cecropins—can be strategically modified to either maintain their helical conformation for membrane interaction or undergo transitions to coil or extended conformations that disrupt β-sheet stacking in amyloid aggregates [47,134]. This structural adaptability, particularly the ability to shift between folded and disordered states, enables dynamic interactions with amyloidogenic intermediates and contributes to aggregation inhibition. In addition, macrocyclization and backbone stapling techniques have been used to rigidify peptides into bioactive conformations with enhanced proteolytic resistance and selective binding [146]. Stapled α-helical peptides derived from AMP scaffolds have demonstrated both anti-aggregation activity and improved BBB permeability [147].

Altogether, structure-based engineering provides a critical link between sequence-level design and functional targeting, offering a strategic framework for developing AMP-derived inhibitors capable of modulating amyloid formation with molecular precision. The intrinsic ability of AMP scaffolds to adopt defined secondary structures—combined with their membrane affinity, biocompatibility, and tunable physicochemical properties—positions them as promising platforms for engineering conformation-specific amyloid inhibitors. Continued advancement in structure-guided approaches, particularly those informed by MD simulations and peptide–fibril docking studies, is expected to substantially expand the therapeutic potential of AMP-based agents in combating amyloid-related diseases.

#### Computational and screening approaches

Computational modeling and in silico screening provide a third pillar for the rational design of AMP-derived amyloid modulators. At the structural level, molecular docking and MD simulations remain powerful tools for elucidating how AMP-derived sequences engage amyloid assemblies at atomic resolution. Docking and structure-based virtual screening offer a first-pass strategy for evaluating how AMP analogs interact with fibril ends, lateral surfaces, or soluble oligomers, identifying preferred binding modes, key contact residues, and approximate binding-energy estimates based on shape and electrostatic complementarity [148]. Such studies have been used to screen libraries of helical or β-hairpin peptides against Aβ fibril models, pinpointing sequences that preferentially cap fibril ends or destabilize interstrand β-sheet stacking [149]. These docked poses can then be refined by MD simulations at coarse-grained or all-atom resolution to probe binding stability, conformational rearrangements, and induced fit, including the helix-to-coil or coil-to-β transitions that are characteristic of AMP-derived scaffolds [150]. Long-timescale or enhanced-sampling simulations (e.g., replica-exchange MD or metadynamics) can further map the free-energy landscape associated with fibril disruption, co-aggregate formation, and early nucleation events [151,152].

To move beyond case-by-case docking, however, cross-seeding prediction must be formulated explicitly as a pairwise learning problem: the relevant object is not an AMP or an amyloid sequence in isolation, but an AMP–amyloid pair with a specific modulation outcome (e.g., inhibitory, promoting, neutral, or toxicity-shifting). Current computational tools operate largely in silos: they predict either antimicrobial potency (e.g., CAMP-R3 [120,121] and AMPScanner [153]) or amyloid propensity (e.g., ZipperDB [154], Tango [155], and Waltz [156]), but rarely the interaction between the two. Consequently, building a dedicated “cross-seeding predictor” therefore requires moving from single-sequence classification to pairwise interaction modeling, analogous to protein–protein docking scoring functions but specialized for disordered and aggregating systems. Because a large-scale, standardized dataset of AMP–amyloid interactions is not yet available to train deep neural networks end-to-end, the immediate path forward is physics-guided feature engineering that encodes the mechanistic principles outlined in the “Central mechanisms of cross-seeding” and “AMP-centric design principles of cross-seeding with amyloids” sections. To succeed, ML models should integrate descriptors for both partners and/or their putative interface, rather than relying solely on single-sequence AMP or amyloid propensity predictors. Existing ML tools for antimicrobial activity—such as AMPlify [119], CAMP-R3 [120,121], and DeepAMP [122]—and for amyloid/aggregation propensity (e.g., Aggrescan [125], Tango [155], and Waltz [156]) provide a useful starting point, but a dedicated cross-seeding predictor will require richer feature sets aligned with the proposed AMP-centric design axes.

Conceptually, 4 families of features are particularly important. (a) AMP-side descriptors capture the AMP-centric design axes: net charge and charge density, anisotropic charge patterning (e.g., clustering of Lys/Arg on one face), hydrophobic moment and amphiphilicity indices, β- and helix-propensity profiles, predicted disorder and conformational plasticity, exposure and spacing of aggregation-prone motifs, and simple multivalency proxies such as the presence of flexible cationic tails or self-association tendencies. (b) Amyloid-partner descriptors include global and local charge distribution, intrinsic aggregation-prone segments and predicted steric-zipper cores, surface hydrophobic and electrostatic patterns, and baseline fibril nucleation/elongation propensities. (c) Pairwise/interface-level descriptors go a step further by quantifying compatibility between AMP β-hotspots and amyloid spine segments (e.g., sequence or structural similarity scores), approximate docking or coarse-grained binding energies, overlap of hydrophobic/charged patches at candidate interfaces, and simple indicators of potential directional asymmetry (e.g., whether “AMP-on-amyloid” templating is more favorable than “amyloid-on-AMP”). (d) Contextual descriptors encode the fact that cross-seeding is strongly environment-dependent: membrane-binding propensity, pH and ionic-strength sensitivity, and the presence of cofactors such as glycosaminoglycans or microbial amyloids can be represented as additional features or as separate “tasks” in a multi-task model.

Given the limited and heterogeneous experimental data on AMP–amyloid cross-seeding, we envisage that transfer learning and multi-task architectures will be necessary. Large sequence- or graph-based neural networks pretrained on general AMP or amyloid datasets could be fine-tuned on curated AMP–amyloid pairs with annotated outcomes, using the above feature classes as explicit inputs or as inductive biases. Pairwise encoders that jointly embed AMP and amyloid sequences—such as Siamese or cross-attention architectures—are natural candidates for learning low-dimensional representations of “compatible” versus “incompatible” cross-seeding interfaces. In parallel, generative models and reinforcement-learning frameworks could use these learned scoring functions as objectives to propose new AMP variants that optimize a multi-objective profile: potent antimicrobial activity, desired modulation of a given amyloid target (e.g., strong inhibition of fibril growth without stabilizing toxic oligomers), and acceptable physicochemical properties. In this way, ML does not simply replicate existing AMP or amyloid predictors, but operationalizes the AMP-centered mechanistic design space defined in the “LL-37: A prototypical dual modulator” and “AMP-centric design principles of cross-seeding with amyloids” sections into a quantitative, pairwise screening tool for engineered cross-seeding modulators.

Taken together, the integration of computational screening with high-throughput experimental validation offers a powerful, synergistic framework for the rational design of AMP-derived amyloid inhibitors. While in silico approaches—such as molecular docking, MD, and ML—enable rapid prediction of binding interfaces, aggregation propensity, and structural adaptability, experimental techniques provide the necessary functional readouts to validate and refine these predictions. Platforms such as peptide microarrays, aggregation inhibition assays, and surface plasmon resonance allow for efficient screening of designed variants, generating critical feedback that can be used to iteratively retrain computational models and improve structural hypotheses. This closed-loop design paradigm enhances predictive accuracy, reduces development time, and enables the systematic optimization of peptide therapeutics. As datasets expand and algorithms improve, the seamless integration of computational and experimental tools will be instrumental in accelerating the discovery of effective, conformation-specific anti-amyloid agents.

### Translational and therapeutic potential of engineered AMPs

The convergence of antimicrobial and amyloid-modulating properties in engineered peptides offers a compelling therapeutic opportunity, particularly for complex diseases that lie at the intersection of protein misfolding, chronic inflammation, and microbial dysregulation. AMPs naturally possess structural features—such as amphipathicity, β-sheet or α-helical conformations, and membrane affinity—that can be harnessed to selectively target amyloidogenic species. Recent progress in sequence-guided and structure-based peptide engineering has enabled the development of AMP derivatives that inhibit amyloid aggregation, neutralize toxic oligomers, and modulate inflammatory cascades [112,133]. These multifunctional peptides are uniquely positioned for translational applications in neurodegenerative disorders, systemic amyloidoses, and amyloid-associated inflammatory conditions. However, their successful clinical deployment depends on overcoming several key translational barriers, including optimization of pharmacological properties, enhancement of delivery efficiency, and assurance of therapeutic safety and selectivity.

#### Therapeutic applications across amyloid diseases

Engineered AMPs—particularly those capable of adopting β-hairpin or amphipathic structures—have shown solid promise in inhibiting amyloid nucleation and fibril elongation. Several AMP families, including LL-37 [53], PG-1 [31], and defensins [35], as well as their engineered analogs [133], exhibit sequence-independent inhibitory activity across diverse amyloid targets, supporting the feasibility of broad-spectrum anti-amyloid strategies. Owing to their dual action on protein aggregation and immune signaling pathways, AMP-based therapeutics are well-suited to address the multifactorial nature of neurodegenerative pathologies. Furthermore, their endogenous origins may confer lower immunogenicity compared to monoclonal antibodies or synthetic inhibitors.

Beyond the CNS, AMP-derived inhibitors also hold strong potential in the treatment of systemic amyloidoses such as AA amyloidosis, TTR amyloidosis, and T2D, where amyloidogenic proteins like serum amyloid A, TTR, and hIAPP deposit in peripheral tissues. Several AMP-inspired β-sheet breakers and peptide mimetics have demonstrated efficacy in inhibiting hIAPP fibril formation and in preserving β-cell function in diabetic models [140]. Given the conserved structural architecture among amyloid fibrils across tissues, engineered AMPs may serve as cross-reactive inhibitors capable of targeting multiple amyloid species using a single scaffold. In addition to primary amyloidosis, amyloid-like aggregates and pro-inflammatory intermediates are increasingly recognized in chronic inflammatory and infectious conditions—including IBD, chronic liver disease, and microbial infections—where bacterial curli or host-derived amyloids contribute to immune dysregulation [157]. AMPs that simultaneously inhibit amyloid formation and modulate innate immune responses represent a unique class of therapeutic agents capable of addressing both proteotoxicity and inflammation, particularly in disorders driven by microbe-associated or sterile protein misfolding triggers.

#### Drug-like optimization of AMP-derived modulators

To enable clinical translation, AMP-derived inhibitors must be engineered to exhibit enhanced pharmacological properties, most notably improved proteolytic stability, conformational selectivity, and systemic safety. A range of chemical and structural optimization strategies have been developed to enhance peptide performance while preserving their biological activity. Stability enhancements are essential for maintaining peptide bioactivity under physiological conditions. Common approaches include the incorporation of D-amino acids to resist protease degradation while preserving functionally relevant conformations. Additionally, backbone cyclization and macrocyclization are used to constrain peptide flexibility and protect terminal residues from enzymatic cleavage. Modifications such as PEGylation or lipidation further increase circulation time, reduce renal clearance, and improve overall pharmacokinetics [158]. Selectivity optimization plays a central role in reducing off-target effects and ensuring therapeutic efficacy. Structure-guided design enables precise tuning of physicochemical features—such as charge distribution, hydrophobicity, and amphipathicity—to favor binding to pathogenic amyloid conformers over native protein structures [159]. The inclusion of β-sheet recognition domains or aggregation-prone motifs can further enhance selectivity for misfolded targets. Advances in targeted delivery systems, including nanoparticles and ligand-modified conjugates, have improved tissue-specific accumulation and on-target engagement [160]. To support systemic safety, AMP-derived candidates are increasingly developed using rational design principles that minimize unintended membrane disruption and immune activation. This includes truncation of membrane-active domains, refinement of amphipathic balance, and the use of endogenous human peptide templates—such as LL-37 and β-defensins—to improve tolerability and immunocompatibility. Early-phase screening for hemolysis, cytokine release, and PRR activation is also essential in identifying safe and therapeutically viable leads. Together, these strategies allow AMP-derived inhibitors to achieve drug-like profiles suitable for in vivo applications.

#### Drug delivery and pharmacokinetics

Effective delivery and pharmacokinetic tuning are fundamental to realizing the therapeutic potential of AMP-based amyloid inhibitors, particularly in diseases involving central or systemic amyloid deposition. The physicochemical nature of peptides—including limited membrane permeability, susceptibility to proteolysis, and rapid clearance—requires tailored delivery approaches. In CNS applications, the BBB poses a major obstacle to therapeutic access. To address this, several strategies have been developed. These include conjugation to BBB-shuttle peptides such as TAT [161], Angiopep-2 [162], and transferrin receptor ligands that mediate receptor-driven transcytosis [163]. Intranasal delivery is also under investigation as a noninvasive route that bypasses the BBB via olfactory and trigeminal pathways [164]. Encapsulation of AMP-derived inhibitors in liposomes, polymeric micelles, or nanoparticles can enhance CNS stability and prolong residence time within target tissues [165,166].

For systemic amyloidosis, delivery strategies focus on improving peptide stability in circulation and enhancing tissue accumulation at sites of amyloid deposition. PEGylation [167] or hydrophilic surface [168] modification can reduce opsonization and clearance, while organ- or disease-specific targeting ligands facilitate selective biodistribution [169]. These formulations not only improve on-target efficacy but also reduce immunogenicity and systemic toxicity. Pharmacokinetic modulation through controlled-release platforms, depot injections, or hydrogel-based delivery systems can further sustain therapeutic exposure. ADME (absorption, distribution, metabolism, and excretion) profiling [170] and real-time imaging [171] are increasingly used to guide formulation decisions and optimize dosing regimens. Together, these delivery and pharmacokinetic approaches are essential to advancing AMP-derived inhibitors from conceptual design to clinical application across both CNS and peripheral disease settings.

## Conclusions and Outlook

AMPs or engineered AMPs that modulate amyloid aggregation represent an emerging and conceptually innovative therapeutic strategy for addressing amyloid-associated diseases. These peptides uniquely combine structural adaptability, innate immune functionality, and tunable physicochemical properties, enabling them to intervene at the intersection of protein misfolding, inflammation, and host defense. Recent advances in rational design, supported by structural modeling, computational screening, and experimental validation, have yielded AMP derivatives capable of inhibiting amyloid aggregation, neutralizing toxic intermediates, and attenuating downstream immune responses in preclinical models of both neurodegenerative and systemic amyloidoses.

Despite this growing promise, several challenges must be addressed to enable successful clinical translation. A major gap lies in the incomplete understanding of the molecular features that govern dual antimicrobial and anti-amyloid functionality. While ML and structural prediction tools have been applied to each domain separately, their integration remains limited, and curated dual-function datasets are still scarce. In parallel, effective delivery—particularly across the BBB—remains a key barrier for CNS indications, and peptide stability and immunogenicity continue to pose challenges even with D-amino acid substitution, cyclization, or conjugation strategies. Variability in in vitro aggregation assays, toxicity measurements, and disease models further hampers reproducibility and cross-comparability across studies. Ultimately, advancing this field will require moving beyond phenomenological observations to address a focused set of high-value questions, including the following:

1. Can we define a minimal, mechanistically grounded descriptor set that predicts when a given AMP will behave as an inhibitor, a neutral binder, or a cross-seeding co-aggregator for a specific amyloid target, and—within that space—is it possible to structurally decouple the membrane-puncturing activity required for antimicrobial defense from the amyloid-capping or remodeling activity needed for neuroprotection? Resolving this tension is essential for avoiding off-target neurotoxicity while preserving innate immune competence.
2. Should the therapeutic objective be the brute-force suppression of aggregation, or is it more effective in some contexts to harness cross-seeding to “trap” toxic oligomers into inert, antimicrobial co-aggregates—i.e., to design directed co-assembly rather than simple inhibition? This question challenges the traditional inhibitor paradigm and demands rigorous in vivo readouts of both aggregation state and host defense.
3. How can we quantitatively measure AMP exposure, half-life, and cross-seeding activity in vivo—particularly in the CNS—at physiologically relevant concentrations and timescales, and what delivery and stabilization strategies best preserve the desired heterotypic interfaces while minimizing toxicity and immunogenicity?
4. Can we design “smart”, conditional modulators that are quiescent in the periphery but become structurally activated only within the acidic, oxidative, or inflammatory microenvironments of plaques, infected tissues, or biofilms, and in which clinical settings (prophylactic, early disease-modifying, or as part of combination regimens) would such agents provide clear added value over existing treatments?

Addressing these questions will require multi-objective ML platforms that integrate aggregation modulation, antimicrobial efficacy, pharmacokinetics, and safety; advances in peptide synthesis and formulation for scalable, cost-effective manufacturing; and coordinated efforts to standardize experimental models, in vivo imaging, and data sharing. In conclusion, AMP-derived amyloid modulators offer a compelling therapeutic modality with mechanistic versatility and modular design potential. Focused progress on the key questions above, supported by close collaboration between peptide chemists, computational biologists, immunologists, and translational scientists, will be pivotal in overcoming existing barriers and realizing the full potential of these next-generation multifunctional therapeutics.

## Supplementary Materials

Supplementary 1 Graphical Abstract
