# Supplementary figures and images for: Antimicrobial Peptides as Cross-Seeding Modulators at the Neurodegenerative–Infectious Interface
Source: Research (Wash D C). 2026 Feb 24;9:1149. doi: 10.34133/research.1149 (PMC12930082; doi:10.34133/research.1149)

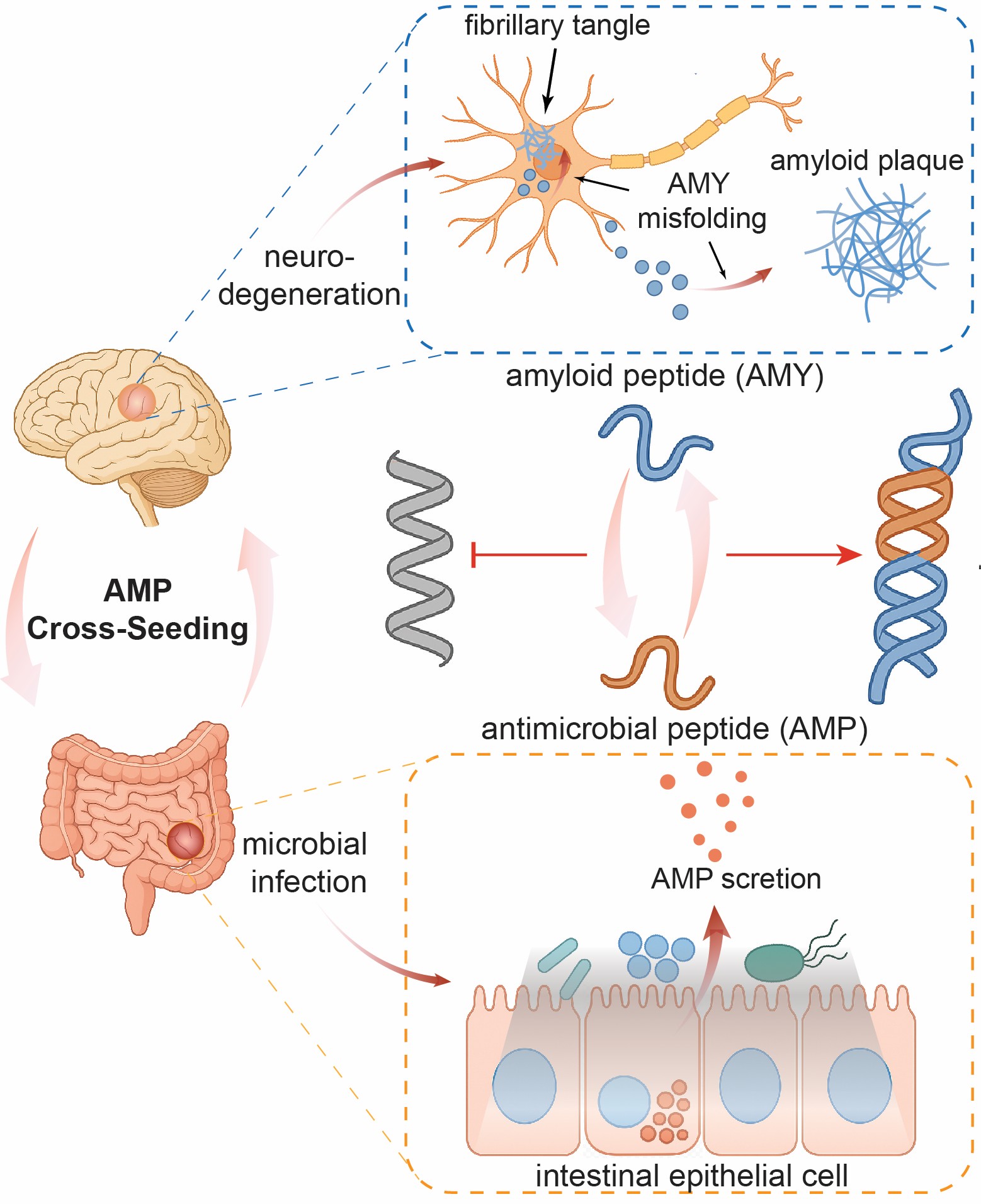

Supplement: Supplementary 1 — Graphical Abstract [file research.1149.f1.zip › table of content.jpg]
